# LRRK1-mediated NDEL1 phosphorylation promotes cilia disassembly via dynein-2-driven retrograde intraflagellar transport

**DOI:** 10.1242/jcs.259999

**Published:** 2022-11-04

**Authors:** Hiroshi Hanafusa, Shin Kedashiro, Mako Gotoh, Ko-hei Saitoh, Hironori Inaba, Tomoki Nishioka, Kozo Kaibuchi, Masaki Inagaki, Naoki Hisamoto, Kunihiro Matsumoto

**Affiliations:** ^1^ Division of Biological Science, Graduate School of Science, Nagoya University, Chikusa-ku, Nagoya, 464-8602, Japan; ^2^ Department of Physiology, Graduate School of Medicine, Mie University, Tsu, Mie, 514-8507, Japan; ^3^ Research Project for Neural and Tumor Signaling, Institute for Comprehensive Medical Science, Fujita Health University, Toyoake, Aichi, 470-1192, Japan

**Keywords:** LRRK1, NDEL1, Primary cilia

## Abstract

Primary cilia are antenna-like organelles that regulate growth and development via extracellular signals. However, the molecular mechanisms underlying cilia dynamics, particularly those regulating their disassembly, are not well understood. Here, we show that leucine-rich repeat kinase 1 (LRRK1) plays a role in regulating cilia disassembly. The depletion of LRRK1 impairs primary cilia resorption following serum stimulation in cultured cells. Polo-like kinase 1 (PLK1) plays an important role in this process. During ciliary resorption, PLK1 phosphorylates LRRK1 at the primary cilia base, resulting in its activation. We identified nuclear distribution protein nudE-like 1 (NDEL1), which is known to positively regulate cilia disassembly, as a target of LRRK1 phosphorylation. Whereas LRRK1 phosphorylation of NDEL1 on Ser-155 promotes NDEL1 interaction with the intermediate chains of cytoplasmic dynein-2, it is also crucial for triggering ciliary resorption through dynein-2-driven retrograde intraflagellar transport. These findings provide evidence that a novel PLK1–LRRK1–NDEL1 pathway regulates cilia disassembly.

## Teaser

**Highlighted Article:** Phosphorylation of NDEL1 Ser-155 by LRRK1 promotes binding of NDEL1 to the dynein-2 intermediate chains, which is required for cilia disassembly via dynein-2-mediated retrograde intraflagellar transport.

## INTRODUCTION

Primary cilia are evolutionarily conserved microtubule (MT)-based organelles protruding from the surface of most mammalian cells (Satir and Christensen, 2007; Ishikawa and Marshall, 2011). The primary cilium originates from the basal body, a modified mother centriole that comprises the centrosome along with the daughter centriole and pericentriolar material. The centrosome functions as an MT-organizing center during mitotic division. During interphase, the centrosome translocates to the plasma membrane and the mother centriole templates the nucleation of the axoneme, the MT-based structural core of the cilium. The primary cilium serves as a specialized subcellular compartment that concentrates membrane-bound receptors and acts as a cellular ‘antenna’ for sensing and responding to the extracellular environment (Singla and Reiter, 2006; Nachury and Mick, 2019). Thus, primary cilia are sensory cellular organelles that play a crucial role in regulating cellular signaling pathways required for cell division and differentiation. The physiological significance of primary cilia for human health has been highlighted by the association of ciliary dysfunction with several human genetic diseases, including developmental defects, obesity and polycystic kidney disease (Gerdes et al., 2009; Goetz and Anderson, 2010; Hildebrandt et al., 2011; Reiter and Leroux, 2017).

The formation of the primary cilium is dynamically regulated, with assembly occurring during cell cycle exit and disassembly coinciding with cell cycle re-entry (Pugacheva et al., 2007; Kim and Tsiokas, 2011). Several mitotic kinases, such as Aurora A and polo-like kinase 1 (PLK1), have been implicated in the coordination between ciliary disassembly and cell cycle progression (Pugacheva et al., 2007; Inoko et al., 2012; Plotnikova et al., 2012; Wang et al., 2013). These kinases are known to play various roles in mitosis, such as mitotic entry, regulation of G2/M checkpoint, centrosome maturation, and spindle assembly (Nigg, 2001; Taylor and Peters, 2008). They are also activated in response to cell cycle re-entry cues to phosphorylate and activate several downstream components required for cilia disassembly. Aurora-A and PLK1 enhance the activity of histone deacetylase 6 (HDAC6), which induces ciliary MTs de-acetylation and disassembly (Pugacheva et al., 2007; Wang et al., 2013). PLK1 also phosphorylates and activates Kif2a, a member of the kinesin-13 family of de-polymerizing kinesins, to promote the de-polymerization of ciliary MTs and disassembly of primary cilia following serum re-stimulation (Miyamoto et al., 2015). From this perspective, identifying a novel regulator of primary cilia could improve understanding of the molecular mechanisms underlying primary cilia disassembly.

We have previously demonstrated that LRRK1 regulates the orientation of mitotic spindles and that LRRK1 is a PLK1 substrate (Hanafusa et al., 2015). PLK1 phosphorylation is required for the CDK1-mediated activation of LRRK1 at the mitotic centrosome. This process regulates the mitotic spindle orientation by nucleating the growth of astral MTs from the centrosomes. LRRK1 is related to the familial Parkinsonism gene product Park8 (also known as LRRK2) and belongs to the ROCO family of proteins, which contain a Ras of complex proteins (ROC) GTPase domain and a MAPKKK-like kinase domain (Bosgraaf and Van Haastert, 2003). LRRK1 localizes to centrosomes throughout the cell cycle (Hanafusa et al., 2015). However, the role of LRRK1 in the centrosomes during interphase remains unclear. Interestingly, the pathogenic LRRK2 mutant has recently been reported to suppress cilia formation in cell culture and the mouse brain (Steger et al., 2017; Dhekne et al., 2018). Given these results, we sought to determine whether LRRK1 is also involved in ciliogenesis in interphase centrosomes.

Cilia are assembled and maintained by the process of intraflagellar transport (IFT), which is mediated by two large multisubunit cargo-binding complexes, IFT-A and IFT-B (Taschner and Lorentzen, 2016). Both the IFT-A and IFT-B complexes undergo kinesin-2-driven motility from the base to the tip, where the complexes are then reorganized before retrograde transport by dynein-2 from the tip to the base (Hou and Withman, 2015; Jensen and Leroux, 2017). The role of dynein-2 has been extensively studied in Chlamydomonas, *Caenorhabditis elegans*, and mice. In all cases, loss of the dynein-2 heavy chain (DHC) results in short, stumpy cilia that accumulate IFT particles at the tip, consistent with the role of dynein-2 in retrograde ciliary transport (Hou and Witman, 2015; Vuolo et al., 2020). The spatial and temporal control of dynein function relies on its regulation by the non-catalytic subunits of the dynein complex, as well as adaptor proteins (Roberts, 2018; Canty and Yildiz, 2020). The dynein accessory protein, nuclear distribution protein nudE-like 1 (NDEL1), has been implicated in regulating dynein motor and/or cargo-binding activities (Pandey and Smith, 2011; Torisawa et al., 2011; Kuijpers et al., 2016).

In this study, we found that LRRK1 is required for serum-induced cilia disassembly and identified NDEL1 as a substrate of LRRK1 kinase during cilia disassembly. The phosphorylation of NDEL1 Ser-155 by LRRK1 promotes NDEL1 binding to the dynein-2 intermediate chains and is necessary for ciliary resorption through dynein-2-mediated retrograde IFT. Our findings suggest that the LRRK1–NDEL1 axis plays a role in regulating cilia disassembly.

## RESULTS

### LRRK1 is required for serum-induced ciliary resorption

**Fig. 1.**
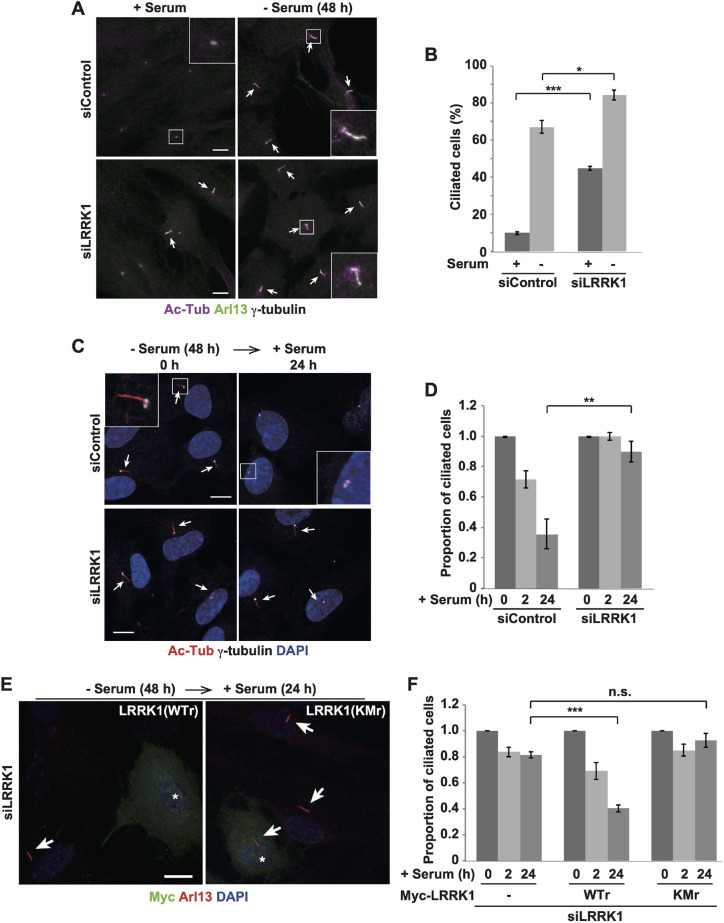
**LRRK1 is required for serum-induced ciliary resorption.** (A,B) Effect of LRRK1 depletion on ciliation. Immunofluorescence images (A) and the percentage of ciliated cells (B) are shown. RPE1 cells were treated with control siRNA or LRRK1-specific siRNA. After incubation with or without serum for 48 h, cells were immunostained with antibodies against Ac-Tub (magenta) and Arl13b (green) for cilia and γ-tubulin (gray) for centrosomes (*n*=3; >200 cells counted per condition). Arrows indicate the primary cilia. The boxed regions are magnified in insets. Scale bars: 10 µm. Percentages of ciliated cells (B) were quantified and are shown as mean±s.d. *P<0.05; ***P<0.001 compared with the control (one-way ANOVA with Tukey's post test). (C,D) Effect of LRRK1 depletion on serum-induced ciliary resorption. Immunofluorescence images (C) and the proportion of ciliated cells (D) are shown. RPE1 cells were treated with control siRNA or LRRK1 siRNA. After 48 h of serum starvation, cells were incubated with serum for the indicated times, and immunostained with antibodies against Ac-Tub (red) and γ-tubulin (gray). DAPI (blue) was used to label cell nuclei (*n*=3; >200 cells counted per condition). Arrows indicate the primary cilia. The boxed regions are magnified in insets. Scale bars: 10 µm. The graph shows the proportion of ciliated cells at each time relative to+serum 0 h, which is set to 1 (D). The proportion of ciliated cells was quantified and is shown as mean±s.d. **P<0.01 (one-way ANOVA with Tukey's post test). (E,F) LRRK1 kinase activity is required for serum-induced ciliary resorption. Immunofluorescence images (E) and the proportion of ciliated cells (F) are shown. RPE1 cells treated with LRRK1 siRNA were transfected with siRNA-resistant wild-type Myc–LRRK1 (WTr) or a kinase-negative Myc–LRRK1 (KMr). After 48 h of serum starvation, cells were incubated with serum for the indicated times, and immunostained with antibodies against Myc (green) and Arl13b (red). DAPI (blue) was used to label cell nuclei (*n*=3; >100 cells counted per condition). Asterisks indicate Myc–LRRK1(WTr or KMr)-expressing cells. Arrows indicate the primary cilia. Scale bar: 10 µm. The graph shows the proportion of ciliated cells at each time relative to +serum 0 h, which is set to 1, as mean±s.d. (F). The percentage of ciliated cells at +serum 0 h was: – Myc–LRRK1, 76±2.8%; WTr, 61±3.8%; KMr, 69±2.5%. Results represent mean±s.d. ***P<0.001; n.s., not significant compared with siLRRK1 24 h (one-way ANOVA with Tukey's post test).

To investigate the role of LRRK1 in ciliogenesis, we assessed the effect of LRRK1 depletion on ciliogenesis in human telomerase-immortalized retinal pigment epithelial cells RPE1 (hTERT-RPE1) cells, which are commonly used for studying cilia (Pugacheva et al., 2007). RPE1 cells were immunostained with antibodies against acetylated α-tubulin (Ac-Tub) and Arl13b, which label cilia (Pugacheva et al., 2007; Cantagrel et al., 2008). We also used an antibody against γ-tubulin to visualize the basal body (mother centriole) and daughter centriole (Fig. 1A). As observed previously (Pugacheva et al., 2007), a large fraction (>65%) of RPE1 cells developed primary cilia upon serum withdrawal (Fig. 1A,B). When RPE1 cells were transfected with LRRK1 siRNA (Fig. S1A,B) and serum-starved for 48 h, the depletion of LRRK1 in RPE1 cells increased the percentage of ciliated cells (Fig. 1A,B). Furthermore, compared to the control, LRRK1 knockdown resulted in a significant increase in ciliation in the presence of serum (Fig. 1A,B), probably due to cell cycle arrest, suggesting that LRRK1 is not required for primary cilia formation.

Proper cilium formation is regulated by the balance between assembly and disassembly (Wang and Dynlacht, 2018). Next, we investigated whether LRRK1 contributes to primary cilia disassembly. RPE1 cells were serum-starved for 48 h, to allow the cells to generate primary cilia, and then stimulated with serum to induce cilia disassembly. In control siRNA-treated cells, RPE1 cells resorbed primary cilia after serum stimulation (Fig. 1C,D). In contrast, only a small fraction of RPE1 cells treated with LRRK1 siRNA resorbed primary cilia during the same period (Fig. 1C,D). These results suggest that LRRK1 is required for the proper disassembly of primary cilia after serum stimulation. To determine whether the kinase activity of LRRK1 is required for its ability to promote cilia disassembly, we used an LRRK1 kinase-negative mutant, in which Lys-1243 in the kinase domain was replaced with methionine [LRRK1(KM)] (Hanafusa et al., 2011). In LRRK1-depleted cells, expression of siRNA-resistant wild-type Myc–LRRK1, but not siRNA-resistant Myc–LRRK1(KM), rescued the defect in cilia disassembly (Fig. 1E,F). These results indicate that the kinase activity of LRRK1 is crucial during cilia disassembly.

### LRRK1 is activated at the ciliary base during cilia resorption

**Fig. 2.**
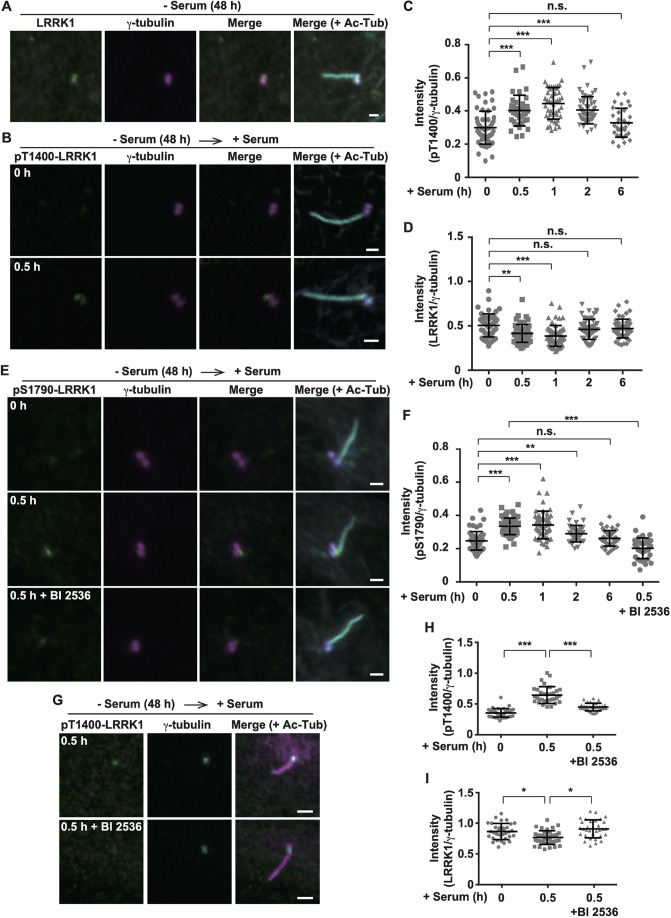
**LRRK1 activated at the ciliary base regulates serum-induced ciliary resorption.** (A) Localization of LRRK1. After 48 h of serum starvation, RPE1 cells were immunostained with antibodies against LRRK1 (green), γ-tubulin (magenta) and Ac-Tub (cyan). Scale bar: 1 µm. (B) LRRK1 activation at the ciliary base. After 48 h of serum starvation, RPE1 cells were incubated with serum for 0.5 h, and immunostained with antibodies against pT1400-LRRK1 (green), γ-tubulin (magenta), and Ac-Tub (cyan). Scale bars: 1 µm. (C,D) Quantification of the fluorescence intensity ratio of pT1400-LRRK1/γ-tubulin signals (C) or LRRK1/γ-tubulin signals (D). After 48 h of serum starvation, RPE1 cells were incubated with serum for 0, 0.5, 1, 2 or 6 h. Immunostaining was performed as described in B except for the antibodies used. Data were plotted as the ratio of pT1400-LRRK1/γ-tubulin signals or LRRK1/γ-tubulin signals (*n*=3; >30 cells counted per condition) with mean±s.d. shown. **P<0.01; ***P<0.001; n.s., not significant (Dunnett's multiple-comparison test). (E) S1790 phosphorylation of endogenous LRRK1. After 48 h of serum starvation, RPE1 cells were treated with or without BI 2536 (100 nM) for 3 h, incubated with serum for 0.5 h, and immunostained with antibodies against pS1790-LRRK1 (green), γ-tubulin (magenta) and Ac-Tub (cyan). Scale bars: 1 µm. (F) Quantification of the fluorescence intensity ratio of pS1790-LRRK1/γ-tubulin signals. After 48 h of serum starvation, RPE1 cells were treated with or without BI 2536 (100 nM) for 3 h and incubated with serum for 0, 0.5, 1, 2, or 6 h. Immunostaining was performed as described in E. Data were plotted as the ratio of pS1790-LRRK1/γ-tubulin signals (*n*=3; >30 cells counted per condition) with mean±s.d. shown. **P<0.01; ***P<0.001; n.s., not significant (Dunnett's multiple-comparison test). (G) Effect of BI 2536 on the pT1400-LRRK1 signal. After 48 h of serum starvation, RPE1 cells were treated with or without BI 2536 (100 nM) for 3 h, incubated with serum for 0.5 h, and immunostained with antibodies against pT1400-LRRK1 (green), γ-tubulin (cyan), and Ac-Tub (magenta). Scale bars: 1 µm. (H,I) Quantification of the fluorescence intensity ratio of pT1400-LRRK1/γ-tubulin signals (H) or LRRK1/γ-tubulin signals (I). Immunostaining was performed as described in G except for the antibodies used. Data were plotted as the ratio of pT1400-LRRK1/γ-tubulin signals or LRRK1/γ-tubulin signals (*n*=3; >30 cells counted per condition) with mean±s.d. shown. *P<0.05; ***P<0.001. (Dunnett's multiple-comparison test).

Next, we investigated the spatiotemporal activation pattern of endogenous LRRK1 during ciliary resorption. To monitor endogenous LRRK1 activation, we used an anti-pT1400 LRRK1 antibody that specifically recognizes the active form of LRRK1, which is phosphorylated on Thr-1400 (Hanafusa et al., 2015). Staining of RPE1 cells with an anti-LRRK1 antibody revealed that LRRK1 colocalized with γ-tubulin in both the mother (basal body) and daughter centrioles (Fig. 2A). The pT1400-LRRK1 signal was barely visible in serum-starved RPE1 cells (Fig. 2B), but LRRK1 Thr-1400 phosphorylation was predominantly observed at the ciliary base adjacent to the mother centriole transiently 0.5–2 h after serum treatment (Fig. 2B,C). The intensity of the LRRK1 signal was slightly reduced 0.5–1 h after serum addition (Fig. 2D). These results suggest that LRRK1 is activated at the ciliary base during cilia resorption.

We previously demonstrated that PLK1 phosphorylates LRRK1 on Ser-1790 and this phosphorylation is required for the activation of LRRK1 at mitotic centrosomes (Hanafusa et al., 2015). Given that PLK1 is involved in cilia disassembly (Lee et al., 2012; Seeger-Nukpezah et al., 2012; Wang et al., 2013; Miyamoto et al., 2015), we assessed the contribution of PLK1 to the regulation of LRRK1 during cilia disassembly. PLK1 localizes to the transition zone at the ciliary base and is activated immediately after serum-induced cilia disassembly (Seeger-Nukpezah et al., 2012; Miyamoto et al., 2015). We determined whether PLK1 is required for serum-induced activation of LRRK1 at the base of primary cilia. First, we investigated whether LRRK1 Ser-1790 phosphorylation occurs during cilia disassembly using an anti-pS1790 LRRK1 antibody (Hanafusa et al., 2015). Immunofluorescence staining of RPE1 cells revealed that the pS1790-LRRK1 signal increased at the ciliary base transiently 0.5–2 h after serum stimulation (Fig. 2E,F). When the serum re-stimulated cells were treated with BI 2536, a PLK1 inhibitor (Lénárt et al., 2007; Steegmaier et al., 2007), the pS1790-LRRK1 signal was reduced to basal levels (Fig. 2E,F). Thus, PLK1-mediated phosphorylation of LRRK1 Ser-1790 is dispensable for its localization at the ciliary base. These results indicate that PLK1 at the ciliary base phosphorylates LRRK1 at Ser-1790 during cilia disassembly following serum re-stimulation.

PLK1 phosphorylation of LRRK1 Ser-1790 triggers the subsequent phosphorylation of Thr-1400 by CDK1, leading to the activation of LRRK1 at centrosomes during mitosis (Hanafusa et al., 2015). The inhibition of PLK1 activity by BI 2536 caused a marked reduction in pT1400-LRRK1 staining at the ciliary base in serum-treated cells (Fig. 2G,H). However, BI 2536 treatment produced a weak effect on LRRK1 protein levels at the ciliary base (Fig. 2I). Thus, the pS1790-LRRK1 signal showed similar temporal and spatial localization to that of pT1400-LRRK1. These results suggest that LRRK1 is phosphorylated on Ser-1790 by PLK1 and is activated at the base of primary cilia during disassembly.

### LRRK1 phosphorylates NDEL1 on Ser-155 at the base of primary cilia

**Fig. 3.**
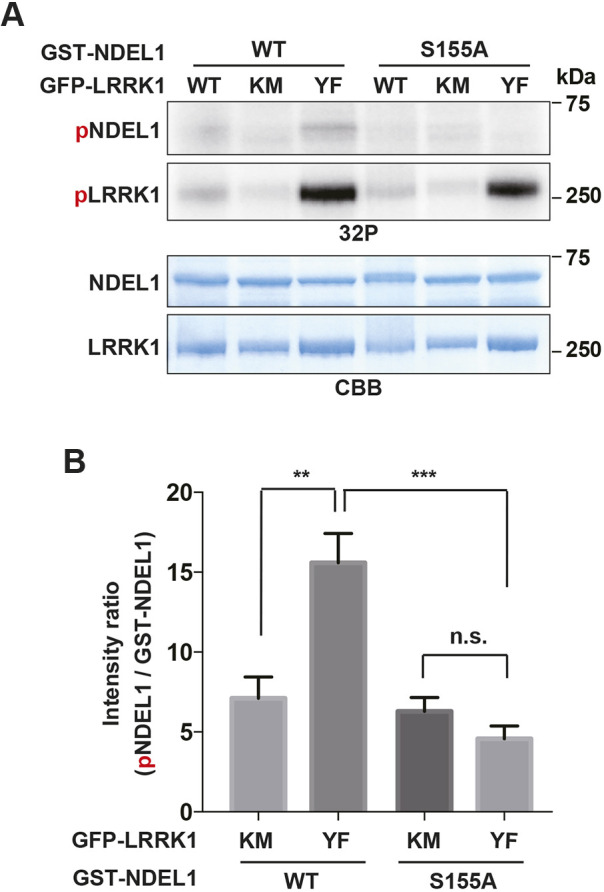
**LRRK1 phosphorylates NDEL1 on Ser-155 *in vitro*.** (A) LRRK1 phosphorylates NDEL1 at Ser-155 *in vitro*. HEK293 cells were transfected with GFP–LRRK1 [WT, KM or LRRK1(Y944F) (YF) mutants], and cell lysates were immunoprecipitated with the anti-GFP antibody. The immuno-purified LRRK1 proteins were incubated with recombinant GST–NDEL1 (WT or S155A mutant) in the presence of [γ-^32^P] ATP for 20 min at 30°C. Autophosphorylated LRRK1 and phosphorylated NDEL1 were resolved by SDS-PAGE (^32^P). Protein input was confirmed by Coomassie Brilliant Blue (CBB) staining. (B) Relative levels of phosphorylation by LRRK1. The levels of phosphorylated NDEL1 were normalized to those of total GST-NDEL1 with mean±s.d. shown. Data are combined from three independent experiments. **P<0.01; ***P<0.001; n.s., not significant (Dunnett's multiple-comparison test).

We next determined the target of LRRK1 phosphorylation that regulates cilia disassembly. We have previously reported that NDEL1 plays an important role in ciliary absorption upon cell cycle re-entry (Inaba et al., 2016). Therefore, we examined whether LRRK1 phosphorylates NDEL1. For this purpose, we performed an *in vitro* kinase assay using glutathione S-transferase (GST)-tagged NDEL1 produced from *Escherichia coli* and GFP–LRRK1 purified from HEK293 cells. We observed phosphorylation of NDEL1 when incubated with the hyper-active LRRK1 mutant LRRK1(Y944F) (Ishikawa et al., 2012), but no phosphorylation when incubated with the kinase-inactive LRRK1(KM) (Fig. 3A,B). These results indicate that LRRK1 phosphorylates NDEL1 *in vitro*. To define the phosphorylation sites on NDEL1, GST–NDEL1 was incubated with LRRK1(Y944F) or LRRK1(KM) as for the *in vitro* kinase assay and analyzed by liquid chromatography-coupled tandem mass spectrometry (LC-MS/MS). The following six phosphorylation sites were assigned: Ser-95, Thr-132, Ser-155, Ser-162, Ser-166, and Ser-213 (Table S1). Within these sites, the Ser-155 site represents the major phosphorylation site for LRRK1, because phosphorylation of the GST–NDEL1(S155A) mutant was greatly reduced in our *in vitro* kinase assay (Fig. 3A,B). Thus, LRRK1 phosphorylates NDEL1 on Ser-155.

**Fig. 4.**
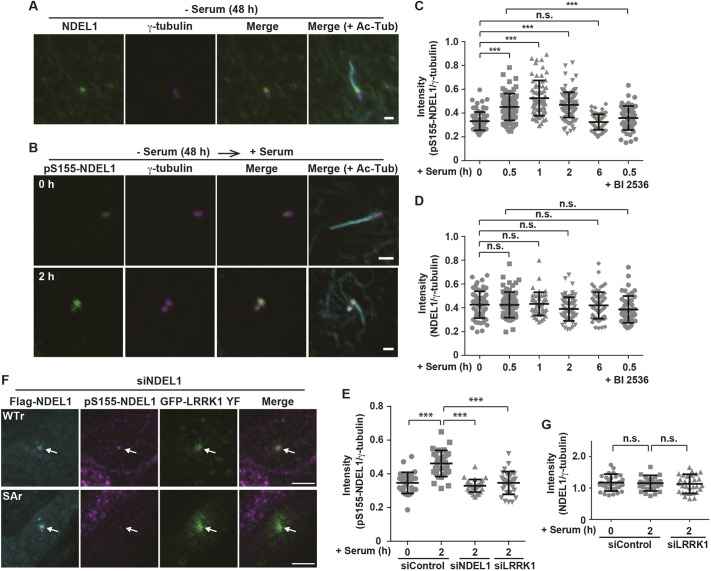
**NDEL1 Ser-155 is phosphorylated by LRRK1 at the ciliary base.** (A) Localization of NDEL1. After 48 h of serum starvation, RPE1 cells were immunostained with antibodies against NDEL1 (green), γ-tubulin (magenta) and Ac-Tub (cyan). Scale bar: 1 µm. (B) Localization of pS155-NDEL1. After 48 h of serum starvation, RPE1 cells were incubated with serum for 2 h, and immunostained with antibodies against pS155-NDEL1 (green), γ-tubulin (magenta) and Ac-Tub (cyan). Scale bars: 1 µm. (C,D) Quantification of the fluorescence intensity ratio of pS155-NDEL1/γ-tubulin signals (C) or NDEL1/γ-tubulin signals (D). After 48 h of serum starvation, RPE1 cells were treated with or without BI 2536 (100 nM) for 3 h and incubated with serum for 0, 0.5, 1, 2 or 6 h. Immunostaining was performed as described in B except for the antibodies used. Data were plotted as the ratio of pS155-NDEL1/γ-tubulin signals or NDEL1/γ-tubulin signals (*n*=3; >30 cells counted per condition) with mean±s.d. shown. ***P<0.001; n.s., not significant (Dunnett's multiple-comparison test). (E) Effect of NDEL1 or LRRK1 depletion on the pS155-NDEL1 signal. RPE1 cells were treated with control siRNA or NDEL1 siRNA, or LRRK1 siRNA. After 48 h serum starvation, cells were incubated with or without serum for 2 h, and immunostained with antibodies against pS155-NDEL1 and γ-tubulin. Data were plotted as the ratio of pS155-NDEL1/γ-tubulin signals (*n*=3; >30 cells counted per condition) with mean±s.d. shown. ***P<0.001 (Dunnett's multiple-comparison test). (F) Specificity of the pS155-NDEL1 antibody. RPE1 cells treated with NDEL1 siRNA were co-transfected with GFP-LRRK1(Y944F) and Flag–NDEL1 (WTr or S155Ar mutant). After 48 h of serum starvation, cells were immunostained with antibodies against Flag (cyan) and pS155-NDEL1 (magenta). Arrows indicate the base of primary cilia. Scale bars: 1 µm. (G) Effect of LRRK1 depletion on the NDEL1 signal. RPE1 cells were treated with control siRNA or LRRK1 siRNA. After 48 h of serum starvation, cells were incubated with or without serum for 2 h, and immunostained with antibodies against NDEL1 and γ-tubulin. Data were plotted as the ratio of NDEL1/γ-tubulin signals (n=3; >30 cells counted per condition) with mean±s.d. shown. n.s., not significant (Dunnett's multiple-comparison test).

We monitored the phosphorylation pattern and localization of endogenous NDEL1 during cilia disassembly. For this purpose, we generated an antibody specific for NDEL1 phosphorylated at Ser-155 (pS155-NDEL1). Immunostaining experiments revealed that NDEL1 was localized predominantly at the base of primary cilia in serum-starved RPE1 cells (Fig. 4A) (Inaba et al., 2016). We found that levels of pS155-NDEL1 at the base of primary cilia increased 0.5–2 h after serum addition (Fig. 4B,C), whereas serum addition did not affect the NDEL1 protein levels at the base of primary cilia (Fig. 4D). We confirmed the specificity of the pS155-NDEL1 antibody. NDEL1 siRNA suppressed the serum-induced increase in signals recognized by the anti-pS155-NDEL1 antibody at the base of primary cilia (Fig. 4E). Furthermore, when constitutively activated GFP–LRRK1(Y944F) was co-expressed with siRNA-resistant wild-type Flag–NDEL1 or non-phosphorylatable NDEL1(S155A) in NDEL1-depleted cells, the signal recognized by the anti-pS155-NDEL1 antibody was detected with wild-type NDEL1 but not in NDEL1(S155A) (Fig. 4F). Thus, the pS155-NDEL1 antibody specifically recognizes Ser-155-phosphorylated NDEL1 at the base of primary cilia.

Next, we examined the effect of LRRK1 or PLK1 inhibition on pS155-NDEL1 levels at the base of primary cilia. When LRRK1 was depleted with siRNA, serum addition did not increase pS155-NDEL1 levels at the base of primary cilia (Fig. 4E,G). Furthermore, treatment of cells with the PLK1 inhibitor BI 2536 reduced pS155-NDEL1 staining at the ciliary base (Fig. 4C,D). These results suggest that the PLK1–LRRK1 pathway induces phosphorylation of NDEL1 Ser-155 at the base of primary cilia.

### Phosphorylation of NDEL1 Ser-155 is required for ciliary disassembly

**Fig. 5.**
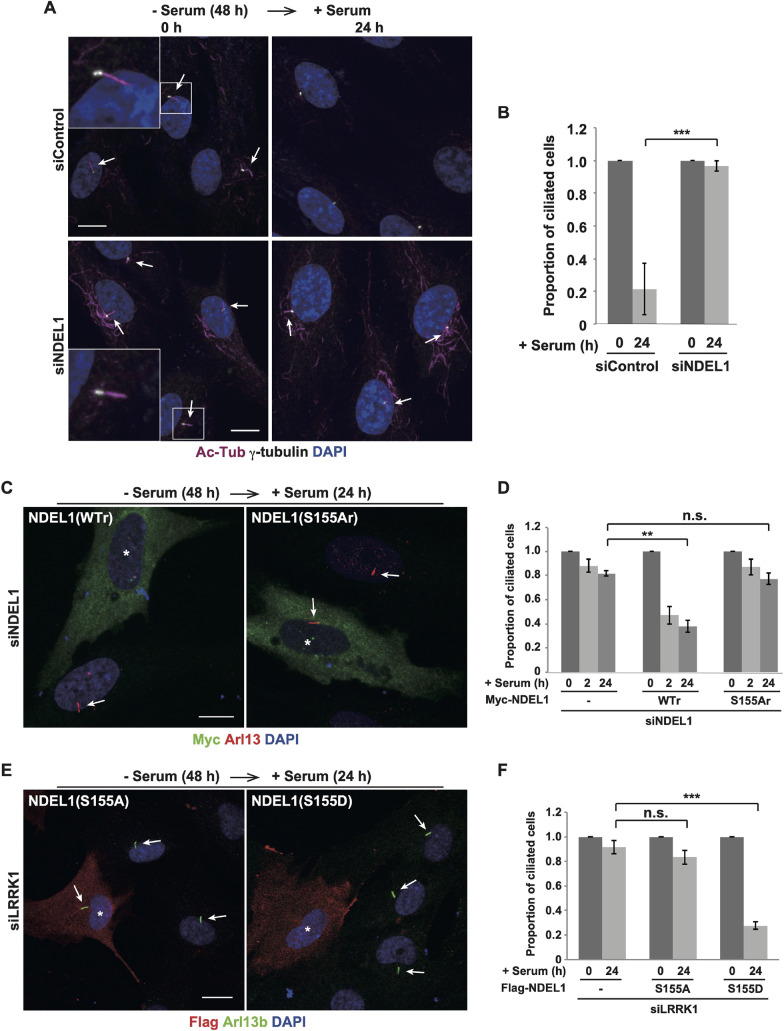
**Phosphorylation of NDEL1 Ser-155 is required for cilia disassembly.** (A,B) Effect of NDEL1 depletion on serum-induced ciliary resorption. Immunofluorescence images (A) and the proportion of ciliated cells (B) are shown. RPE1 cells were treated with control siRNA or NDEL1 siRNA. After 48 h of serum starvation, cells were incubated with serum for the indicated times, and immunostained with antibodies against Ac-Tub (magenta) and γ-tubulin (gray). DAPI (blue) was used to label cell nuclei (*n*=3; >200 cells counted per condition). Arrows indicate the primary cilia. Scale bars: 10 µm. The graph shows the proportion of ciliated cells at each time relative to +serum 0 h, which is set to 1, as mean±s.d. (B).  The percentage of ciliated cells was: +serum 0 h; siControl, 85±5.6%; siNDEL1, 85±2.6%. ***P<0.001 (one-way ANOVA with Tukey's post test). (C,D) Effect of NDEL1 Ser-155 phosphorylation on serum-induced ciliary resorption. Immunofluorescence images (C) and the proportion of ciliated cells (D) are shown. RPE1 cells treated with NDEL1 siRNA were transfected with siRNA-resistant wild-type Myc–NDEL1 (WTr) or a non-phosphorylatable mutant Myc–NDEL1 (S155Ar). After 48 h of serum starvation, cells were incubated with serum for the indicated times, and immunostained with antibodies against Myc (green) and Arl13b (red). DAPI (blue) was used to label cell nuclei (*n*=3; >100 cells counted per condition). Asterisks indicate Myc–NDEL1(WTr or S155Ar mutant)-expressing cells. Arrows indicate the primary cilia. Scale bar: 10 µm. The graph shows the proportion of ciliated cells at each time relative to +serum 0 h, which is set to 1, as mean±s.d. (D). The percentage of ciliated cells at was: +serum 0 h; − Myc–NDEL1, 75±1.8%; WTr, 41±4.4%; S155Ar, 62±2.9%. **P<0.01; n.s., not significant compared with siNDEL1 24 h (one-way ANOVA with Tukey's post test). (E,F) LRRK1 regulates ciliary resorption by phosphorylating NDEL1 Ser-155. Immunofluorescence images (E) and the proportion of ciliated cells (F) are shown. RPE1 cells treated with LRRK1 siRNA were transfected with Flag–NDEL1(S155A or S155D mutants). After 48 h of serum starvation, cells were incubated with serum for the indicated times, and immunostained with antibodies against Flag (red) and Arl13b (green). DAPI (blue) was used to label cell nuclei (*n*=3; >200 cells counted per condition). Asterisks indicate Flag–NDEL1(S155A or S155D mutants)-expressing cells. Arrows show primary cilia. Scale bar: 10 µm. The graph shows the proportion of ciliated cells at each time relative to +serum 0 h, which is set to 1, as mean±s.d. (F). The percentage of ciliated cells at +serum 0 h; − Flag–NDEL1, 84±1.2%; S155A, 74±4.6%; S155D, 65±4.7%. ***P<0.001; n.s., not significant compared with siLRRK1 24 h (one-way ANOVA with Tukey's post test).

Given that NDEL1 is required for cilia disassembly (Inaba et al., 2016), we hypothesized that LRRK1 controls cilia disassembly by phosphorylating NDEL1. To test this possibility, we investigated the relationship between LRRK1 and NDEL1 in cilia disassembly. We confirmed that NDEL1 is necessary for cilia disassembly. RPE1 cells treated with NDEL1 siRNA (Fig. S2) were serum-starved for 48 h and stimulated by the re-addition of serum. We observed that NDEL1 depletion decreased the efficiency of ciliary resorption (Fig. 5A,B). To investigate the functional relevance of NDEL1 Ser-155 phosphorylation in cilia disassembly, we expressed siRNA-resistant wild-type Myc–NDEL1 or the non-phosphorylatable mutant S155A, in NDEL1-depleted cells. We found that wild-type Myc–NDEL1 was able to rescue the defect in ciliary disassembly caused by NDEL1 knockdown, whereas Myc–NDEL1(S155A) was not (Fig. 5C,D). These results suggest that the phosphorylation of NDEL1 Ser-155 is important for cilia disassembly. If LRRK1 regulates cilia disassembly by phosphorylating NDEL1 at Ser-155, the phospho-mimicking mutant NDEL1(S155D) would be expected to compensate for LRRK1 depletion in cilia disassembly. We found that the expression of Flag–NDEL1(S155D), but not Flag–NDEL1(S155A), suppressed the defect in ciliary resorption observed in LRRK1-depleted cells (Fig. 5E,F). These results suggest that LRRK1-mediated phosphorylation of NDEL1 Ser-155 is required for cilia disassembly.

### The LRRK1–NDEL1 pathway regulates cilia disassembly via dynein-2-driven retrograde IFT

**Fig. 6.**
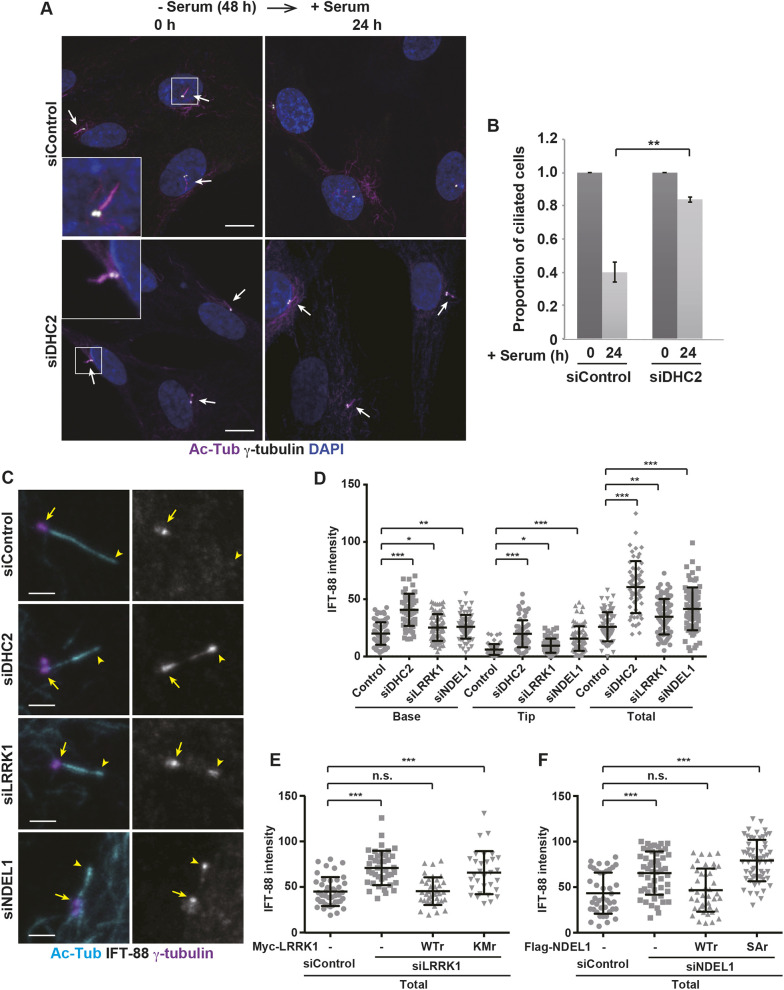
**Effect of DHC2 depletion on serum-induced ciliary resorption and IFT88 localization.** (A,B) DHC2 is required for serum-induced ciliary resorption. Immunofluorescence images (A) and the proportion of ciliated cells (B) are shown. RPE1 cells were treated with control siRNA or DHC2 siRNA. After 48 h of serum starvation, cells were incubated with serum for the indicated times, and immunostained with antibodies against Ac-Tub (magenta) and γ-tubulin (gray). DAPI (blue) was used to label cell nuclei (*n*=3; >200 cells counted per condition). Arrows indicate the primary cilia. The boxed regions are magnified in insets. Scale bars: 10 µm. The graph shows the proportion of ciliated cells at each time relative to +serum 0 h, which is set to 1, as mean±s.d. (B). The percentage of ciliated cells at +serum 0 h; siControl, 85±2.3%; siDHC2, 86±2.0%. **P<0.01 (one-way ANOVA with Tukey's post test). (C,D) Effect of DHC2, LRRK1 or NDEL1 depletion on IFT88 localization. Immunofluorescence images (C) and quantification of IFT88 localization within cilia (D) are shown. RPE1 cells were treated with control siRNA, DHC2 siRNA, LRRK1 siRNA or NDEL1 siRNA. After 48 h of serum starvation, cells were immunostained with antibodies against Ac-Tub (cyan), IFT88 (gray) and γ-tubulin (magenta). Arrowheads and arrows indicate the tip and base of the primary cilia, respectively. Scale bars: 1 µm. Data (D) were plotted for the fluorescence intensity of IFT88 signals (*n*=3; >30 cells counted per condition) with mean±s.d. shown. *P<0.05; **P<0.01; ***P<0.001 (Dunnett's multiple-comparison test). (E) LRRK1 kinase activity is required for IFT88 localization in cilia. RPE1 cells treated with control siRNA or LRRK1 siRNA were transfected with siRNA-resistant Myc–LRRK1 (WTr or KMr mutant) as indicated. After 48 h of serum starvation, cells were immunostained with antibodies against Ac-Tub, IFT88 and γ-tubulin. Data were plotted for the fluorescence intensity of IFT88 signals (*n*=3; >30 cells counted per condition) with mean±s.d. shown. ***P<0.001; n.s., not significant (Dunnett's multiple-comparison test). (F) Phosphorylation of NDEL1 Ser-155 is required for IFT88 localization in cilia. RPE1 cells treated with control siRNA or NDEL1 siRNA were transfected with siRNA-resistant Myc–NDEL1 [WTr or a non-phosphorylatable S155A mutant (SAr)]. After 48 h of serum starvation, cells were immunostained with antibodies against Ac-Tub, IFT88 and γ-tubulin. Data were plotted for the fluorescence intensity of IFT88 signals (*n*=3; >30 cells counted per condition) with mean±s.d. shown. ***P<0.001; n.s., not significant (Dunnett's multiple-comparison test).

How does the phosphorylation of NDEL1 exert its function in ciliary resorption? NDEL1 is an accessory protein of the dynein-1 motor complex, and regulates its motor activity (Pandey and Smith, 2011; Torisawa et al., 2011; Kuijpers et al., 2016). Dynein-2-mediated retrograde transport of ciliary components is thought to be required for ciliary resorption (Hou and Witman, 2015). Based on these findings, we hypothesized that the LRRK1–NDEL1 pathway might promote ciliary resorption by activating dynein-2-driven retrograde IFT within the cilium. We first examined whether dynein-2 is required for cilia disassembly by knocking down dynein heavy chain 2 (DHC2; also known as DYNC2H1). Cilium formation in cells depleted of DHC2 by means of siRNA (Fig. S3) was induced by serum starvation. As observed previously (Palmer et al., 2011), DHC2 siRNA produced shorter cilia compared to the control siRNA (Fig. 6A; Fig. S4). DHC2 siRNA treatment significantly reduced the deciliation process after serum addition (Fig. 6A,B). These results indicate that dynein-2 is indeed required for cilia disassembly.

Given that dynein-2 is the motor for retrograde IFT in cilia (Hou and Witman, 2015; Roberts, 2018; Vuolo et al., 2020), we examined and compared the ciliary localization of IFT88 (a subunit of the IFT-B complex) in cells treated with ciliobrevin, a specific dynein inhibitor. When RPE1 cells were serum-starved for 48 h and then stimulated with serum in the presence of ciliobrevin, the efficiency of ciliary resorption decreased (Fig. S5A). As in previous studies (Hamada et al., 2018; Vuolo et al., 2018; Tsurumi et al., 2019), after 48 h of serum starvation, IFT88 was found almost exclusively at the ciliary base in control cells, whereas IFT88 accumulated at the ciliary tip in ciliobrevin-treated cells (Fig. S5B,C). Thus, acute inhibition of dynein-2 activity in serum-starved conditions results in accumulated IFT material at ciliary tips, which is consistent with the role of dynein-2 in retrograde ciliary transport. However, when DHC2 was depleted with siRNA, IFT88 accumulated at both the ciliary tip and base (Fig. 6C,D).

We next investigated the effect of the loss of LRRK1 or NDEL1 on IFT88 localization in cilia. We found that both LRRK1 siRNA and NDEL1 siRNA resulted in a significant accumulation of IFT88 at the ciliary tip and base (Fig. 6C,D). To test whether the LRRK1-mediated phosphorylation of NDEL1 at Ser-155 is important for IFT88 localization in cilia, we performed rescue experiments. When siRNA-resistant wild-type Myc–LRRK1 or kinase–negative LRRK1(KM) was expressed in LRRK1-depleted cells, wild-type LRRK1, but not LRRK1(KM), rescused abnormal IFT88 accumulation in the cilia (Fig. 6E). Similarly, we found that wild-type Flag–NDEL1 could rescue the defect in IFT88 accumulation caused by NDEL1 knockdown, but NDEL1(S155A) could not (Fig. 6F). These results suggest that the phosphorylation of NDEL1 Ser-155 by LRRK1 is essential for IFT88 trafficking in cilia.

Next, we analyzed the effect of DHC-2, LRRK1 and NDEL1 depletion on the localization of the IFT-A protein, IFT140, in cilia. As observed previously (Hamada et al., 2018; Vuolo et al., 2018), after 48 h of serum starvation, IFT140 was found only at the ciliary base in the control cells (Fig. S6A,B). However, the loss of DHC-2, LRRK1 or NDEL1 had little effect on the ciliary localization of IFT140 (Fig. S6A,B).

### NDEL1 is associated with the dynein-2 intermediate chains

**Fig. 7.**
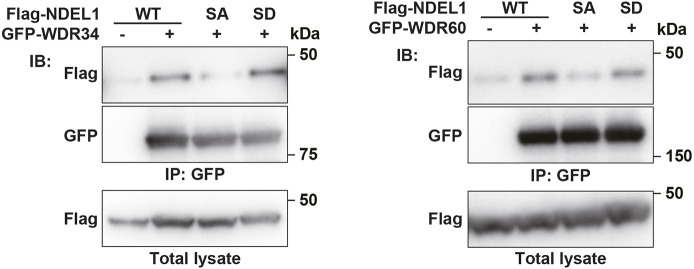
**Ser-155 phosphorylation of NDEL1 is critical for its interaction with dynein-2 intermediate chains.** HEK293 cells were co-transfected with Flag-NDEL1 (WT, S155A, or S155D mutants), and GFP-WDR34 or GFP-WDR60, as indicated. Complex formation was detected by immunoprecipitation (IP) with the anti-GFP antibody, followed by immunoblotting (IB) with the anti-Flag antibody. Total lysates were immunoblotted with antibodies as indicated. Blots shown representative of three repeats.

Even though LRRK1 and NDEL1 are localized almost exclusively at the basal body, how would they affect dynein-2 activity at the ciliary tip from the base? One possibility is that LRRK1 phosphorylation of NDEL1 promotes the ciliary entry of dynein-2 or IFT complex proteins involved in retrograde IFT by interacting with dynein-2. This is consistent with a recent report indicating that in *Caenorhabditis elegans*, loss of WDR-60, a homolog of the dynein-2 intermediate chain WDR60 (also known as DYNC2I1) (Asante et al., 2014), impairs dynein-2 recruitment to cilia and its incorporation onto anterograde IFT trains, reducing retrograde motor availability at the ciliary tip (De-Castro et al., 2021). In fact, this model is compatible with the basal body localization of LRRK1 and NDEL1. Given that NDEL1 binds to the dynein-1 intermediate chain (McKenney et al., 2011), we hypothesized that NDEL1 could also bind to the dynein-2 intermediate chains, WDR34 (also known as DYNC2I2) and WDR60. To test this possibility, we transfected Flag–NDEL1 into HEK293 cells with GFP–WDR34 or GFP–WDR60. We found that NDEL1 bound to WDR34 and WDR60 (Fig. 7). Furthermore, WDR34 and WDR60 were significantly less associated with non-phosphorylatable NDEL1(S155A) than wild-type NDEL1 or the S155D phosphomimetic mutant (Fig. 7). These results indicate that Ser-155 phosphorylation of NDEL1 by LRRK1 is important for its interaction with dynein-2 intermediate chains.

## DISCUSSION

The primary cilium is an MT-based organelle protruding from the cell surface, and plays an important role in regulating signal transduction, cell proliferation and differentiation and embryo development. Great progress has been made in uncovering how regulatory proteins control cilia formation (Satir and Christensen, 2007; Ishikawa and Marshall, 2011; Wang and Dynlacht, 2018). However, in contrast with cilia formation, much less information is known about the mechanisms that underlie ciliary resorption. In this study, we demonstrate that the PLK1–LRRK1 cascade promotes primary cilia disassembly via phosphorylation of NDEL1 after re-stimulation with serum. Our findings provide evidence for a link between LRRK1 and NDEL1 and implicate these molecules in the regulation of cilia disassembly via dynein-2-mediated retrograde IFT. We have also previously shown that NDEL1 suppresses ciliogenesis in proliferating cells by regulating the trichoplein–Aurora A pathway (Inaba et al., 2016). Therefore, NDEL1 regulates ciliogenesis by activating dynein-2-driven retrograde IFT in quiescent cells and by regulating the trichoplein–Aurora A pathway in proliferating cells.

We show that LRRK1 regulates serum-induced ciliary resorption in a kinase activity-dependent manner. LRRK1 is activated at the ciliary base upon serum stimulation in quiescent RPE1 cells. What is the mechanism by which its kinase activity is upregulated to promote cilia disassembly? We previously reported that the activation of LRRK1 at mitotic centrosomes depends on the PLK1-mediated phosphorylation of Ser-1790 (Hanafusa et al., 2015). PLK1 is activated at the base of cilia and facilitates ciliary disassembly (Seeger-Nukpezah et al., 2012; Miyamoto et al., 2015). These results raise the possibility that PLK1 is involved in LRRK1 activation during cilia disassembly. Indeed, we found that PLK1 phosphorylates LRRK1 on Ser-1790 at the ciliary base. During mitosis, PLK1-mediated phosphorylation of LRRK1 Ser-1790 leads to the subsequent phosphorylation of Thr-1400 by CDK1, which activates LRRK1 kinase activity at centrosomes (Hanafusa et al., 2015). Consistent with this, we show that treating cells with the PLK1-specific inhibitor BI 2536 reduced the LRRK1 pThr-1400 signal at the ciliary base. These results suggest that PLK1 triggers a cascade of phosphorylation-dependent events that are essential for primary cilia disassembly induced by serum re-stimulation. Most recently, Malik et al. reported that PKC phosphorylates and activates LRRK1 (Malik et al., 2022), but it is as yet unknown whether this phosphorylation is involved in ciliary resorption.

Here, we also identify NDEL1 as a substrate of LRRK1 in regulating cilia disassembly. NDEL1 is phosphorylated on its Ser-155 residue at the base of cilia after serum stimulation in an LRRK1-dependent manner. The phosphorylated NDEL1 on Ser-155 is important for serum-induced ciliary resorption. What function does NDEL1 phosphorylation by LRRK1 play in cilia disassembly? Anterograde transport is dependent on kinesin-2 to carry dynein-2-containing materials to the tip, where new ciliary proteins are incorporated (Taschner and Lorentzen, 2016; Webb, et al., 2020). Retrograde transport, which depends on dynein-2, is responsible for returning the anterograde transport apparatus to the base of the cilium so that it can be recycled (Hou and Witman, 2015; Taschner and Lorentzen, 2016; Webb, et al., 2020). In the IFT model, loss of function of the anterograde motor results in no ciliary growth, and loss of function of the retrograde apparatus causes accumulation of IFT materials at the ciliary tip, producing short and stumpy cilia. However, dynein-2 mutations in mice result in an abnormal accumulation of IFT particles near the ciliary base (Ocbina et al., 2011; Goggolidou et al., 2014). Consistent with this, DHC2 deletion increases IFT88 both at the ciliary tip and near the ciliary base, suggesting that IFT88 could be retained at the basal body or around the transition zone. Indeed, dynein-2 is also required for ciliary transition zone assembly. These results suggest that NDEL1 regulates ciliary resorption via protein sorting and trafficking at the transition zone in a manner dependent on phosphorylation by LRRK1. This possibility could explain why LRRK1 and NDEL1 affect dynein-2 activity from the base to the ciliary tip, even though they are localized almost exclusively at the basal body.

Notably, WDR60 is required for the efficient loading of dynein-2 onto anterograde IFT trains at the ciliary base (De-Castro et al., 2021). Live imaging analysis in *C. elegans* has shown that the loss of WDR60 impairs dynein-2 recruitment to cilia and its incorporation onto anterograde IFT trains, reducing retrograde motor availability at the ciliary tip. Two recent studies in human cells have shown that WDR60 loss leads to the misplacement of IFT and signaling particles in cilia without greatly affecting axoneme extension (Hamada et al., 2018; Vuolo et al., 2018). These results suggest that some IFT subunits might be less efficiently incorporated into anterograde IFT trains in WDR60-deficient cilia. We further demonstrate that NDEL1 interacts with dynein-2 intermediate chains, WDR34 and WDR60 and that these interactions are strengthened by LRRK1-mediated phosphorylation of NDEL1 at Ser-155. Therefore, the mechanism regulating the interaction between NDEL1 and dynein-2 intermediate chains by LRRK1 phosphorylation acts as an important modulator of dynein-2 function. Based on these findings, we propose that the LRRK1–NDEL1 pathway recruits the dynein-2 complex to the ciliary base by interacting with the dynein-2 intermediate chains and promotes dynein-2 loading onto anterograde IFT trains during cilia disassembly. In *Chlamydomonas*, flagellar shortening increases the rate of entry into the flagella of empty IFT trains that possess cargo-binding sites for retrieving disassembled axonemal components from the flagellar tip (Pan and Snell, 2005). However, IFT88 localization does not increase within the cilia during cilium disassembly, suggesting that some mechanistic difference exists between mammalian cilia disassembly and *Chlamydomonas* flagellar shortening.

LRRK1 is related to the familial Parkinsonism gene product Park8 (LRRK2). The pathogenic LRRK2 mutant has recently been reported to function as a negative regulator of primary cilia formation (Steger et al., 2017; Dhekne et al., 2018). LRRK2 blocks ciliogenesis by phosphorylating Rab10, a member of the Rab family of GTPases, and promoting its binding to the effector RILPL1 at pericentriolar membranes (Dhekne et al., 2018). We reported that LRRK1 phosphorylates Rab7 at a conserved serine residue and this increases its interaction with the effector RILP during EGFR–endosome trafficking (Hanafusa et al., 2019). Wang et al. (2019) showed that Rab7a is required for intraciliary F-actin polymerization and is involved in the regulation of ciliary ectocytosis, which is the excision of ciliary tips during cilia disassembly (Phua et al., 2017; Mirvis et al., 2019). It is unknown whether LRRK1 also regulates Rab7-mediated ciliary ectocytosis in addition to NDEL1-mediated cilia disassembly. Further studies will be needed to assess these issues in the future.

## MATERIALS AND METHODS

### Cell cultures, antibodies and reagents

RPE1 cells were cultured in Dulbecco's modified Eagle's medium (DMEM) and F12 nutrient mix (1:1) supplemented with 10% fetal bovine serum (FBS) (042-30555, FUJIFILM Wako). HEK293 cells were cultured in DMEM containing 10% FBS. These cell lines were obtained from the American Type Culture Collection or the Japanese Collection of Research Bioresources and were regularly tested for *Mycoplasma* contamination. Antibodies and their suppliers were as follows: anti-Ac-Tub (T6793, Sigma); anti-γ-tubulin (GTU-88, Sigma); anti-Arl13b (17711-1-AP, Proteintech); anti-IFT88 (13967-1-AP, Proteintech); anti-IFT140 (17460-1-AP, Proteintech); anti-GFP [598, Medical and Biological Laboratories (MBL)]; anti-Flag (M2, Sigma or FLA-1, MBL); anti-Myc (9E10, Santa Cruz Biotechnology). Affinity-purified rabbit antibodies against LRRK1, pT1400-LRRK1, pS1790-LRRK1, and NDEL1 were produced according to a previously described method (Hanafusa et al., 2015; Inaba et al., 2016). A rabbit antibody against pS155-NDEL1 was produced by MBL by injecting rabbits with the synthetic phosphopolypeptide RNAFLEpSELDEKE (where p stands for the phosphorylated residue), coupled to keyhole limpet hemocyanin and affinity purified. BI 2536 and ciliobrevin were purchased from Selleck Chemicals.

### Plasmids, mutations, and RNA interference

Human LRRK1 was cloned from a cDNA library using RT-PCR (our clone lacks 27 amino acids at the N-terminus compared with NM_024652). GFP–LRRK1, GFP–LRRK1(K1243M), and GFP–LRRK1(Y944F) were generated as described previously (Hanafusa et al., 2011; Ishikawa et al., 2012). Human NDEL1 was cloned from a cDNA library using RT-PCR and subcloned into the pCMV-Flag vector (Clontech). NDEL1(S155A) and NDEL1(S155D) were generated using the QuikChange Site-Directed Mutagenesis Kit according to the manufacturer's protocol (Stratagene, La Jolla, CA) and subcloned into the pCMV-Flag vector. siRNA-resistant LRRK1 was generated by mutating the target sequence of the LRRK1 siRNA (5′-GGCCTCGCATTGTATATGA-3′) into 5′-GACCTAGGATCGTCTATGA-3′. siRNA-resistant NDEL1 (WTr or S155Ar) was subcloned into the pDEST-Myc vector (Inaba et al., 2016). siRNA for human LRRK1 [target sequence: 5′-GGCCTCGCATTGTATATGA(TT)-3′], human NDEL1 [target sequence: 5′-GGATATCAGCACTAAACAT(TT)-3′], and human DHC2 [target sequence: 5′-ACAGGCTCTTCTCTCTGAA(TT)-3′] were purchased from Ambion and Life Technologies or JBioS. Control 2 siRNA (Silencer Select; Life Technologies) was used as a negative control. Annealed siRNAs were transfected using RNAiMAX (Invitrogen), and transfected cells were analyzed 72 h after transfection. To estimate the transfection efficiency of siRNA in RPE1 cells, RNA oligonucleotide labeled with Alexa-Fluor-555 was used.

### Preparation of GST fusion proteins and kinase assays

The recombinant proteins GST–NDEL1 and GST–NDEL1(S155A) were each expressed in the *E. coli* strain BL21-CodonPlus (DE3)-RIPL and purified using glutathione-Sepharose 4B (GE Healthcare) following the manufacturer's guidelines. All GFP–LRRK1 proteins were expressed in HEK293 cells and immunopurified with an anti-GFP antibody. Kinase reactions were performed in a final volume of 20 µl buffer containing the following reagents: 50 mM HEPES (pH 7.4), 5 mM MgCl_2_, 5 mM MnCl_2_, 0.5 mM DTT, 5 µCi of [γ-^32^P]ATP, and 100 µM ATP. Samples were incubated for 20 min at 30°C and the reactions were terminated by the addition of Laemmli sample buffer and subsequent boiling. Samples were resolved by SDS-PAGE and analyzed by autoradiography.

### Determination of phosphorylation sites by LC-MS/MS

For analyzing phosphorylation sites, immunoprecipitated proteins were subjected to a non-radioactive *in vitro* kinase assay and then eluted with guanidine solution (50 mM NH_4_HCO_3_, 7 M guanidine-HCl), followed by reduction, alkylation, demineralization and concentration as described previously (Kedashiro et al., 2015). Proteins were digested with trypsin for 16 h at 37°C. From the peptide samples, phosphopeptides were enriched and captured using Titansphere Phos-TiO Kit according to the manufacturer's instructions. Nano-electrospray tandem mass analysis was performed using a Q Exactive mass spectrometer (Thermo Fisher Scientific) system combined with a Paradigm MS4 HPLC system (Michrom BioResources). Samples were injected onto the ADVANCE nanoflow UHPLC/HTS-PAL system equipped with a MonoCap C18 Nanoflow column 0.1 mm×150 mm (GL Sciences). Reversed-phase chromatography was performed with a linear gradient (0 min, 5% B; 70 min, 40% B) of solvent A (H_2_O with 0.1% formic acid) and solvent B (acetonitrile) at an estimated flow rate of 400 nl/min. Ionization was performed with an ADVANCE CaptiveSpray Source (Michrom BioResources Inc.). A precursor ion scan was carried out using a 380–1900 mass-to-charge ratio (*m*/*z*) before MS/MS analysis. Raw data were analyzed using Proteome Discoverer™ software with the Sequest™ algorithm at 15 ppm precursor mass accuracy and 0.02 Da MS/MS tolerance. The peptide search was performed against the UniProtKB *Homo sapiens* reference proteome dataset (release 2012_10) with a 1% false discovery rate cut-off. The determination of the most likely phosphorylation sites was accomplished using the PhosphoRS algorithm within the Proteome Discoverer software.

### Immunoprecipitation

For immunoprecipitation, cells were lysed in RIPA buffer [50 mM Tris-HCl, pH 7.4, 0.15 M NaCl, 0.25% deoxycholic acid, 1% NP-40, 1 mM EDTA, 1 mM dithiothreitol, phosphatase inhibitor cocktail 2 (Sigma) and protease inhibitor cocktail (Sigma)], followed by centrifugation at 15,000* **g*** for 12 min. The supernatant was added to 50 µl (1.5 mg) of Dynabeads Protein G (Invitrogen) with the indicated antibodies and rotated for 2 h at 4°C. The beads were then washed three times with ice-cold phosphate-buffered saline and subjected to immunoblotting. Raw data for western blots shown are provided in Fig. S7.

### Immunofluorescence and image analysis

For immunofluorescence staining, cells were grown on coverslips, treated as indicated, and fixed in 4% paraformaldehyde for 15 min at 37°C or methanol for 2 min at −20°C, permeabilized in 0.5% Triton X-100 for 5 min, and incubated with primary and secondary antibodies. Primary antibodies were as follows: mouse anti-Ac-Tub at 1:5000, anti-γ-tubulin at 1:500, anti-Myc at 1:200, anti-Flag at 1:500, rabbit anti-Arl13b at 1:500, anti-IFT88 at 1: 200, anti-IFT140 at 1:100, anti-LRRK1 at 1:300, anti-pT1400-LRRK1 at 1:250, anti-pS1790-LRRK1 at 1:250, anti-NDEL1 at 1:25,000, and anti-pS155-NDEL1 at 1:500. Secondary antibodies were as follows: Alexa-Fluor 488-, 555- or 647-goat anti-mouse IgG, anti-mouse IgG_2b_, anti-mouse IgG_1_, or anti-rabbit IgG antibodies (Invitrogen). Isotype-specific mouse immunoglobulins were used to colabel Ac-Tub (mouse IgG_2b_) and γ-tubulin (mouse IgG_1_). Confocal microscopy was performed using an Olympus FV3000 microscope. Images were captured at 0.5 µm intervals and *z*-stacks were processed with the FV3000 software. To quantify the signal of LRRK1, pT1400-LRRK1, pS1790-LRRK1, NDEL1, pS155-NDEL1 and γ-tubulin at the base of cilia or basal body, the fluorescence intensity of these molecules was measured and calculated using ImageJ (National Institutes of Health, Bethesda, MD, USA). For the quantification of the IFT88 signal, a circle of the same diameter was drawn at the tip and base of the cilia, respectively, and the fluorescence intensity was measured. Cilium length was determined by Ac-Tub labeling, followed by maximum projection and direct measurement using the ImageJ plugin (National Institutes of Health, Bethesda, MD, USA). To determine cells expressing transiently transfected Myc–LRRK1, Myc–NDEL1 or Flag–NDEL1 at moderate levels, the ImageJ plugin was used to select cells with expression levels below an arbitrary threshold. For each series of experiments, the microscope settings were optimized for the brightest, unsaturated images and remained unaltered during the analysis.

### qPCR analysis

RNA was isolated from cells using the TRIzol extraction method (Invitrogen), and 2 µg of RNA was reverse-transcribed into cDNA using M-MLV reverse transcriptase (Invitrogen) for 60 min at 37°C. Newly synthesized cDNA was then used for real-time qPCR using SYBR Premix Ex Taq^TM^ kit (Takara). The primers used were designed by Takara. The primer pairs (5′–3′) for human DHC2 [(fwd) GAATGTGCCCGCAATGGAG with (rev) TCTGCAGTGAGCCAAAGACGA], and human RPLP1 [(fwd) CCCTGGCCAACGTCAACAT with (rev) TCAGACTCCTCGGATTCTTCTTTCT] were purchased from Takara. Amplification was performed by Applied Biosystems 7300 real-time PCR system. Gene expression was analyzed using the 2(^-ΔΔCT^) method and normalized to RPLP1 as a housekeeping gene. Data were combined from three independent experiments.

### Statistical analysis

Statistical analysis was performed using either Dunnett's multiple-comparison test or Welch's *t*-test. Error bars represent s.d. Detailed *n* values for each panel in figures are stated in the corresponding legends. No statistical method was used to predetermine the sample size. Prism software (GraphPad Software) was used for the analysis of statistical significance.

## Supplementary Material

10.1242/joces.259999_sup1 Supplementary information
