# Supplementary material for: LRRK1-mediated NDEL1 phosphorylation promotes cilia disassembly via dynein-2-driven retrograde intraflagellar transport
Source: J Cell Sci. 2022 Nov 4;135(21):jcs259999. doi: 10.1242/jcs.259999 (PMC9687541; doi:10.1242/jcs.259999)
Supplement: Supplementary information [file joces-135-259999-s1.pdf]

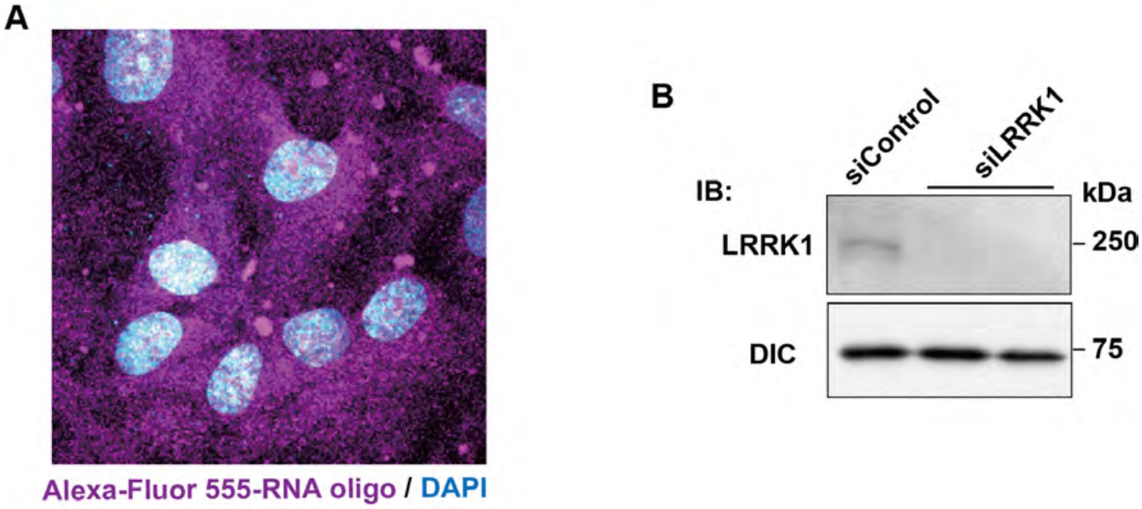


**Fig. S1. Effect of LRRK1 siRNA.**

**(A)** Transfection efficiency of RNA oligo. RPE1 cells were treated with Alexa-Fluor 555-labaled RNA oligo (magenta) and stained with DAPI (gray).

**(B)** RPE1 cells were treated with control siRNA or LRRK1 siRNA. Total lysates were immunoblotted (IB) with antibodies as indicated. Dynein-1 intermediate chain (DIC) is served as the loading control.

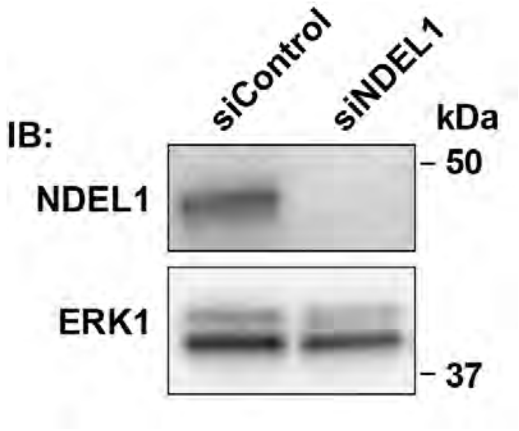


**Fig. S2. Effect of NDEL1 siRNA.**

RPE1 cells were treated with control siRNA or NDEL1 siRNA. Total lysates were immunoblotted (IB) with antibodies as indicated. ERK1 is served as the loading control.

Journal of Cell Science • Supplementary information

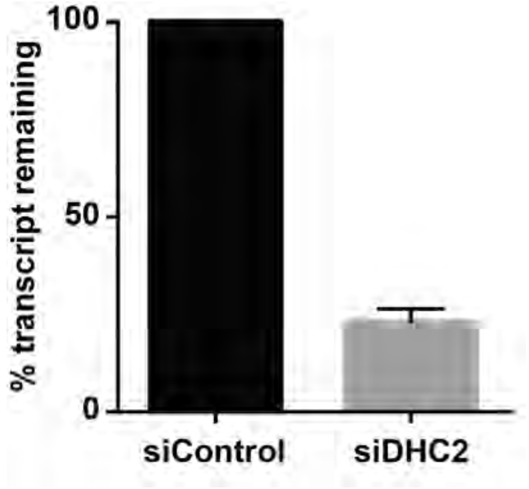


**Fig. S3. Effect of DHC2 siRNA.**

RPE1 cells were treated with control siRNA or DHC2 siRNA. Relative DHC2 mRNA transcript levels were determined by qPCR analysis.

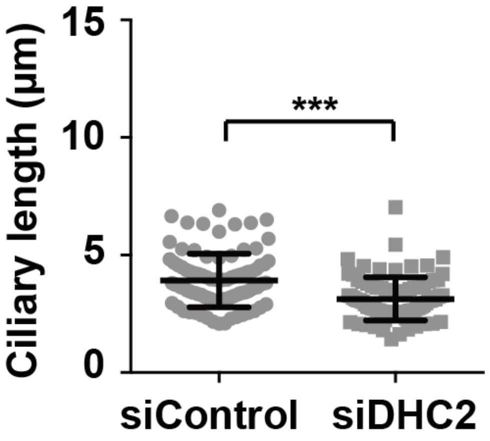


**Fig. S4. Effect of DHC2 siRNA on ciliary length.**

RPE1 cells were treated with control siRNA or DHC2 siRNA. After 48 h of serum starvation, cells were immunostained with antibodies against Ac-Tub (magenta) and γ-tubulin (gray). Ciliary length was quantified using a One-way ANOVA, ****P* < 0.001. Error bars represent s.d.

Journal of Cell Science • Supplementary information

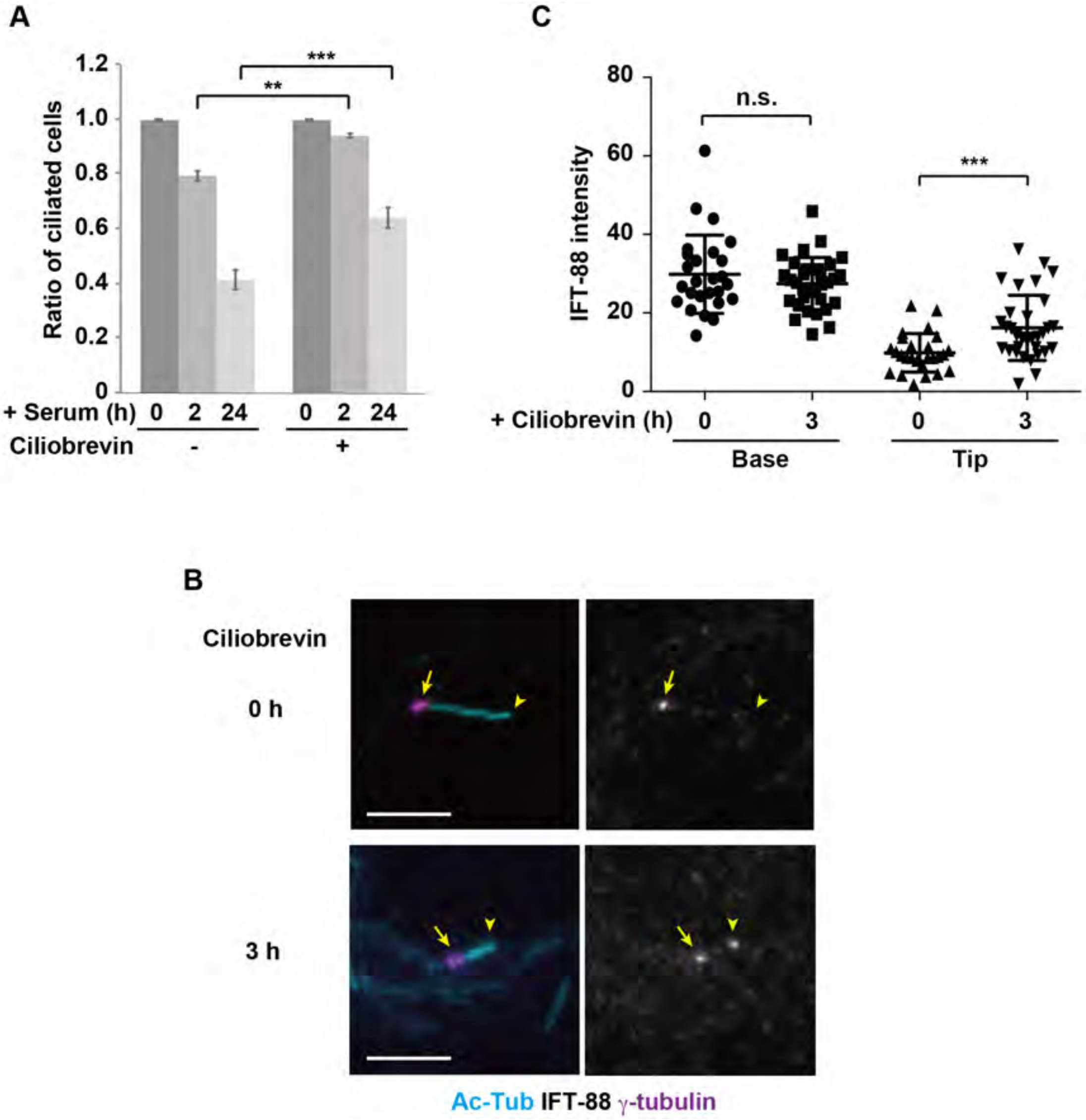


**Fig. S5. Effects of dynein inhibitor on serum-induced ciliary resorption and IFT88 localization.**

**(A)** Effect of a dynein inhibitor on serum-induced ciliary resorption. The ratio of ciliated cells is shown. After 48 h of serum starvation, RPE1cells were treated with or without Ciliobrevin (50 μM) for 1 h, incubated with serum for the indicated times, and immunostained with antibodies against γ-tubulin and Ac-Tub (n = 3; >100 cells counted per condition). The percentages of ciliated cells were quantified using a One-way ANOVA, $^{**}P < 0.01$; $^{***}P < 0.001$. Error bars

represent s.d. The percentage of ciliated cells at + serum 0 h; Control, 85 ± 3.5%; +Ciliobrevin, 84 ± 7.2%.

**(B,C)** Effect of dynein inhibitor on IFT88 localization. Immunofluorescence images **(B)** and quantification of IFT88 localization within the cilia **(C)** are shown. After 48 h of serum starvation, RPE1 cells were treated with or without Ciliobrevin (50 μM) for 3 h, and immunostained with antibodies against Ac-Tub (cyan), IFT88 (gray), and γ-tubulin (magenta). Arrowheads and arrows indicate the tip and base of the primary cilia, respectively. Scale bar: 5 μm. Data **(C)** were plotted for the fluorescence intensity of IFT88 signals (n = 3; >30 cells counted per condition) and quantified using Dunnett's multiple-comparison test. ***$P < 0.001$; n.s., not significant. Error bars represent s.d.

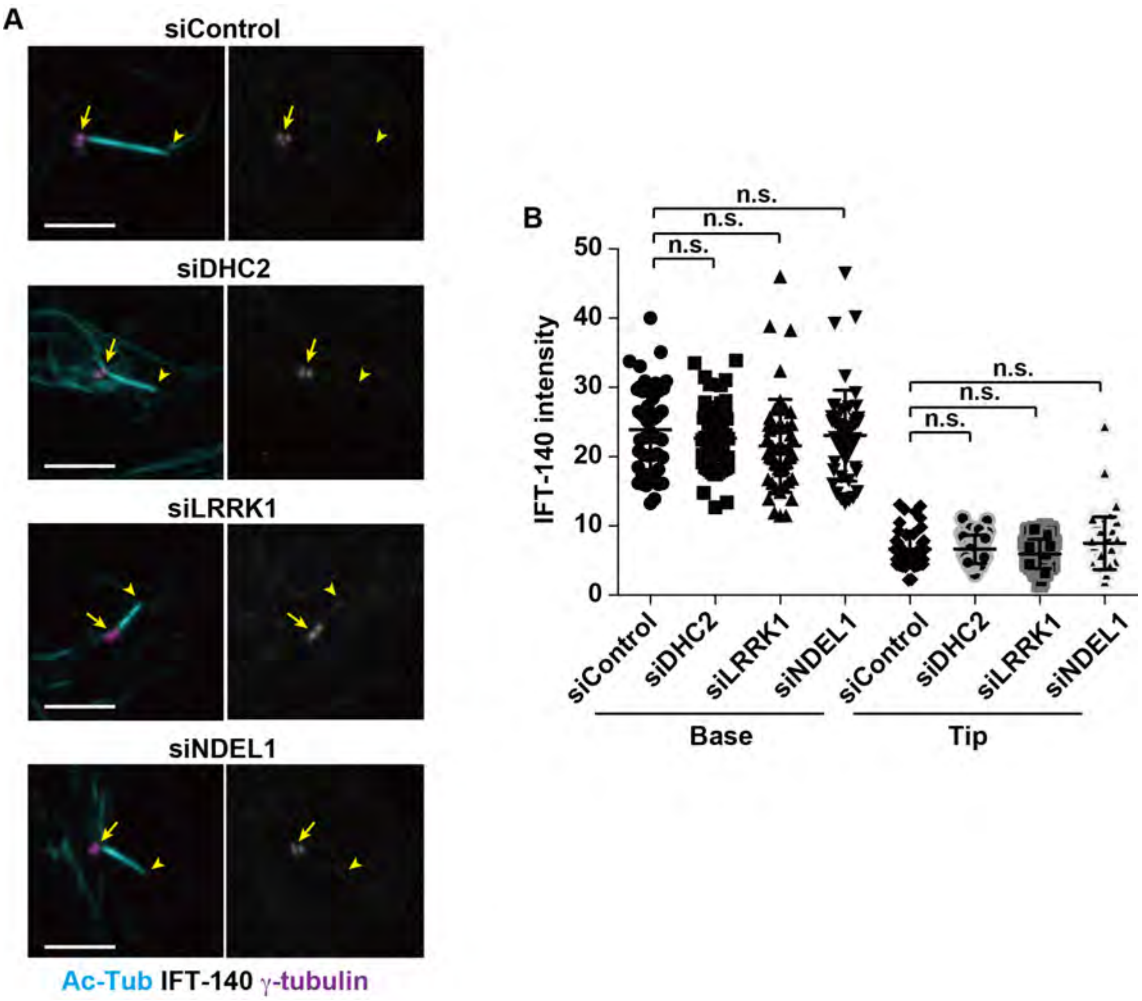


**Fig. S6. Effect of DHC2, LRRK1, or NDEL1 depletion on IFT140 localization.**

**(A,B)** Immunofluorescence images **(A)** and quantification of IFT140 localization within the cilia **(B)** are shown. RPE1 cells were treated with control siRNA, DHC2 siRNA, LRRK1 siRNA, or NDEL1 siRNA. After 48 h of serum starvation, cells were immunostained with antibodies against Ac-Tub (cyan), IFT140 (gray), and γ-tubulin (magenta). Arrowheads and arrows indicate the tip and base of the primary cilia, respectively. Scale bar: 5 μm. Data **(B)** were plotted for the fluorescence intensity of IFT140 signals (n = 3; >30 cells counted per condition) and quantified using Dunnett's multiple-comparison test. n.s., not significant. Error bars represent s.d.

Journal of Cell Science • Supplementary information

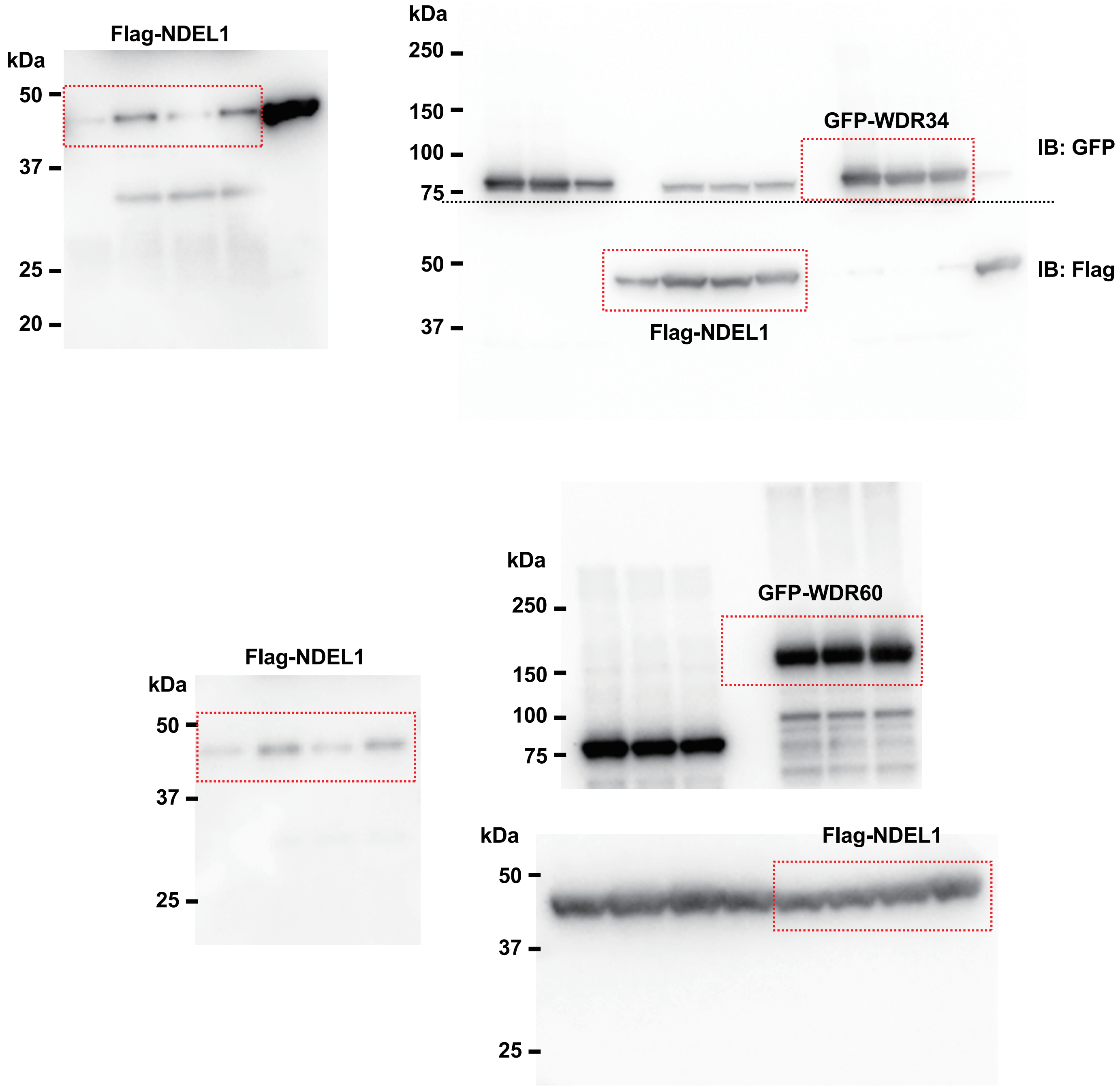


## Fig. S7. Blot transparency

**Related to Figure 7: Uncropped scans of western blots.**

# Table S1. Identification of LRRK1 phosphorylation sites of NDEL1 by LC-MS/MS

| Sequence[a] | Phospho (STY) probabilities[b] | PEP[c] | Identified site(s) |
|---|---|---|---|
| QVp**S**VLEDDLSQTR | S(3): 100.0; S(10): 0.0; T(12): 0.0 | 7.29E-05 | Ser-95 |
| RAp**T**IVSLEDFEQR | T(3): 97.4; S(6): 2.6 | 7.86E-05 | Thr-132 |
| NAFLEp**S**ELDEKESLLVSVQR | S(6): 96.7; S(13): 3.3; S(17): 0.0 | 0.001596 | Ser-155 |
| NAFLESELDEKEp**S**LLVSVQR | S(6): 16.1; S(13): 82.5; S(17): 1.4 | 0.009778 | Ser-162 |
| NAFLESELDEKESLLVp**S**VQR | S(6): 1.1; S(13): 0.9; S(17): 98.0 | 0.010024 | Ser-166 |
| MDSAVQAp**S**LSLPATPVGK | S(3): 0.0; S(8): 87.0; S(10); 12.9: T(14); 0.1 | 4.41E-05 | Ser-213 |

[a]The peptide sequence in the protein. Phosphoseryl and phosphothreonyl residues are denoted as pS and pT, respectively, and are shown in bold.
[b]An estimation of the probability (0-100%) for the respective site being truly phosphorylated.
[c]Posterior error probability of the identification. This value essentially operates as a p-value, where smaller is more significant.
