# Supplementary material for: LRRK1-mediated NDEL1 phosphorylation promotes cilia disassembly via dynein-2-driven retrograde intraflagellar transport
Source: J Cell Sci. 2022 Nov 4;135(21):jcs259999. doi: 10.1242/jcs.259999 (PMC9687541; doi:10.1242/jcs.259999)
Supplement: Supplementary file 1 [file joces-135-259999_review_history.pdf]

Journal of Cell Science

LRRK1-mediated NDEL1 phosphorylation promotes cilia disassembly via dynein-2-driven retrograde intraflagellar transport

Hiroshi Hanafusa, Shin Kedashiro, Mako Gotoh, Ko-hei Saitoh, Hironori Inaba, Tomoki Nishioka, Kozo Kaibuchi, Masaki Inagaki, Naoki Hisamoto and Kunihiro Matsumoto
DOI: 10.1242/jcs.259999

Editor: Michael Way

Review timeline
Original submission: 7 March 2022
Editorial decision: 8 April 2022
First revision received: 6 September 2022
Editorial decision: 27 September 2022
Second revision received: 4 October 2022
Accepted: 5 October 2022

Original submission

First decision letter

MS ID#: JOCES/2022/259999

MS TITLE: The PLK1-LRRK1-NDEL1 axis regulates cilia disassembly via dynein-2-driven retrograde intraflagellar transport

AUTHORS: Hiroshi Hanafusa, Shin Kedashiro, Mako Gotoh, Ko-hei Saitoh, Hironori Inaba, Tomoki Nishioka, Kozo Kaibuchi, Masaki Inagaki, Naoki Hisamoto, and Kunihiro Matsumoto
ARTICLE TYPE: Research Article

We have now reached a decision on the above manuscript.

To see the reviewers' reports and a copy of this decision letter, please go to: https://submit-jcs.biologists.organd click on the 'Manuscripts with Decisions' queue in the Author Area. (Corresponding author only has access to reviews.)

As you will see, all three reviewers are broadly positive but still raise a number of criticisms that prevent me from accepting the paper at this stage. They suggest, however, that a revised version might prove acceptable, if you can address their concerns. If you think that you can deal satisfactorily with the criticisms on revision, I would be pleased to see a revised manuscript. We would then return it to the reviewers.

Please ensure that you clearly highlight all changes made in the revised manuscript. Please avoid using 'Tracked changes' in Word files as these are lost in PDF conversion.

I should be grateful if you would also provide a point-by-point response detailing how you have dealt with the points raised by the reviewers in the 'Response to Reviewers' box. Please attend to all of the reviewers' comments. If you do not agree with any of their criticisms or suggestions please explain clearly why this is so.

© 2022. Published by The Company of Biologists under the terms of the Creative Commons Attribution License (https://creativecommons.org/licenses/by/4.0/).

Reviewer 1

Advance summary and potential significance to field

This manuscript reports the novel observation that depletion of LRRK1 influences cilia loss from cells upon serum addition. It proposes a possible pathway to explain this but a great deal of additional data are needed to support the pathway proposed.

Comments for the author

In 2011, Hanafusa et al. reported that GFP-LRRK1 localizes to early endosomes and regulates trafficking of EGFR. That is generally consistent with the major substrate of LRRK1 being Rab7, a late endosomal protein. They also reported that LRRK1 was needed for peripheral endosomes to be translocated to the perinuclear region. In 2015, Hanafusa et al. reported that LRRK1 is now instead localized exclusively to centrosomes throughout the cell cycle and may be phosphorylated by CDK1/cyclin B in vitro at position T1400. [Which is it? Please clarify with localization data here--for example Fig 1 and 2 are different and many micrographs are very hard to see]. The authors used this finding to conclude that this modification "activates" LRRK1 but this was not shown and is a premature conclusion. The authors previously reported association of PLK1 with LRRK1 in mitosis and transphosphorylation at LRRK1 S1790. It is not yet clear how this modification influences LRRK1 activity or localization. It is also odd that this phosphosite has not been detected in multiple other lab's phosphoproteomics according to Phosphosite.com.

Here the authors would like to conclude that LRRK1 is needed for ciliary disassembly, and they propose a series of phosphorylation steps that they believe drive this: LRRK1 pSer1790 by PLK1, followed by LRRK1 phosphorylation of NDEL1 at Ser155. Inaba et al. (2016) showed that Ndel1 is a suppressor of primary cilia assembly likely through the stabilization of trichoplein at the mother centriole. Here the authors claim that LRRK1 is "stimulated" at the mother centriole in response to serum re-stimulation but they have not yet shown this directly (page 5 line 14).

There is no question that this manuscript reports a great deal of work but many of the conclusions are truly premature and need better documentation. For example, the specificity of the phospho-specific antibodies is not included [can a LRRK1 inhibitor be used? Please show full blots of whole cell extracts and knockouts to demonstrate specificity]. It was not stated how the authors knew which cells had received the siRNAs (even what percentage) and how they knew what they were scoring in the rescue experiments or how much the rescue constructs were over-expressed. Also, given the prior connections to cell division, it is possible that the blockade observed is more related to a cell cycle arrest than a direct link to cilia retraction, especially if the authors need to score phenotypes after such long times of serum starvation.

Specific comments.

Page 7. Line 10. "To monitor LRRK1 activation we used an anti-pT1400 LRRK1 antibody". It has not been shown that this form is actually "activated".

Page 7 Line 21. "...this [Ser1790] phosphorylation is required for the activation of LRRK1 at mitotic centrosomes. Again, the consequence of this modification is not yet known.

Page 8 Line 14 It may be true that CDK1 may phosphorylate Thr1400 after Ser 1790 but again, the consequence of these events is not yet established.

Figure 4 seeks to convince the reader that GFP-LRRK1 binds NDEL1b and not NDEL1a. The inputs do not appear equal and this reader could not determine if the blots were by LICOR or ECL--if ECL, they cannot be compared due to non-linearity of the method. The authors need to determine the significance of this interaction by generating a mutant that cannot bind and determining the consequences of a rescue with that protein. Phosphorylation of NDEL1 is modest in vitro with large amounts of kinase and needs to be determined in cells at endogenous levels of enzyme and substrate. pS155 antibody needs to be properly validated to be sure it does not also recognize non-phospho or other site phospho NDEL1.

© 2022. Published by The Company of Biologists under the terms of the Creative Commons Attribution License (https://creativecommons.org/licenses/by/4.0/).

For all of these reasons, the story needs additional work before it is ready for presentation. It does appear that loss of LRRK1 increases the percent of ciliated cells but this may simply be due to a cell cycle arrest. The authors should use flow cytometry of cells with and without LRRK1 to check this possibility.

Reviewer 2

Advance summary and potential significance to field

In the manuscript by Hanafusa et al, the authors focus on dissecting the role of LRRK1, PLK1 and Ndel1 in ciliary remodeling (specifically resorption subsequent to serum addition). The authors make several very interesting findings, including that LRRK1, PLK1, and Ndel1 are all important for this process. Moreover, they do an excellent job refining the specific phospho-sites that are likely responsible for this activity, and use phospho-specific antibodies to show that serum addition indeed leads to increased phosphorylation of Ndel1.

The data are of high quality, and the findings are very interesting. Among the most interesting of the findings the data suggest that Ndel1 may somehow affect dynein-2 activity. The previous body of literature mostly suggests that Ndel1 functions in regulating dynein-LIS1 interplay. This would then suggest that Ndel1 affects the ability of LIS1 to affect dynein-2 function. There is very little evidence that LIS1 (or Ndel1 for that matter) affects dynein-2 function, although at least one study suggested as much, and found that LIS1 localized to motile (bot not non-motile) mammalian cilia (Pederson et al., JCS 2007). Thus, the work in this manuscript raises the possibility that these well characterized regulators of dynein-1 may also affect dynein-2. I find the study to be of sufficiently high quality to be published in JCS after at least some minor revisions, and potentially an additional experiment or two that may increase its impact (described below).

Comments for the author

Major points

1) Although the authors' findings are indeed provocative and clear, it is unclear to me how Ndel1 might affect dynein-2 activity from the ciliary base (i.e., distal from where dynein-2 functions)? I think potential mechanisms should at least be discussed.

2) The authors' reasoning for focusing on Ndel1 is a bit unclear to me. They previously identified NudC as an LRRK1 binding partner. Why then follow-up with Ndel1 instead of NudC? Did the authors look into a potential role for NudC in ciliary function?

3) Given the fact that Ndel1 binds to the unstructured N-terminus of the dynein-1 intermediate chain (McKenney et al., JBC 2011), the authors' findings suggest that Ndel1 might also bind to the N-terminus of the dynein-2 ICs (WRD60 or WRD34) and thereby affect its activity. This possibility should be discussed along with a description of any conservation between the IC N-termini.

4) Along these same lines, it is fairly well established that Ndel1 affects dynein-1 activity by affecting the dynein-LIS1 interaction. A previous study found that LIS1 may in fact localize to the cilium (Pederson et al., JCS 2007); however, there has been no follow-up or functional assessment of LIS1 in ciliary function.
Although not a requirement for publication, I think it would be very interesting to simply test the role of LIS1 in ciliary resorption (and perhaps IFT88 transport, as shown in Figure 8). If LIS1 depletion does not cause the same phenotype as Ndel1 depletion, this would suggest a LIS1-independent function for Ndel1, which would be interesting. If Ndel1 does function via LIS1, then I would expect depletion of one or the other to cause similar phenotypes, whereas combined depletion should not be additive in severity.

Minor point:

1) "Our findings provide evidence for a link between LRRK1 and NDEL1 and implicate in the regulation of cilia disassembly via dynein-2-mediated retrograde IFT."

© 2022. Published by The Company of Biologists under the terms of the Creative Commons Attribution License (https://creativecommons.org/licenses/by/4.0/).

This sentence is confusingly written. I think the authors meant: “Our findings provide evidence for a link between LRRK1 and NDEL1 and implicate these molecules in the regulation of cilia disassembly via dynein-2-mediated retrograde IFT.”

Reviewer 3

Advance summary and potential significance to field

In this manuscript Hanafuse et al. demonstrate that LRRK1 plays an important role in regulating cilia disassembly, and they provide evidence indicating that PLK1, a known regulator of cilia disassembly, phosphorylates LRRK1 at the base of the cilium to activate it. Once activated, LRRK1 phosphorylates the dynein regulator NDEL1, and this phosphorylation is ultimately what drives the cilium disassembly by a proposed mechanism involving NDEL1-mediated activation of dynein-2 driven retrograde IFT. Overall, this is a nice well-written manuscript that provides new mechanistic insight into the important process of cilia disassembly. Results are generally of high quality, and with a few exceptions (see below), the authors’ claims are well supported by the data. If the authors can address the comments listed below, I think this manuscript will be suitable for publication in Journal of Cell Science.

Comments for the author

Main comments

Figure 7 and 8, p. 11-12 and Discussion: the authors show that DHC2-depleted cells fail to deciliate after 24 hr of serum re-addition (Fig. 7) and based on data indicating that IFT88 accumulates at the tip and base of these cells, as well as in cells depleted for LRRK1 or NDEL1 (Fig. 8), they propose that PLK1/LRRK1/NDEL1 promote cilia disassembly by activating dynein 2-mediated retrograde IFT at the ciliary tip. However, I find that this model is not well supported by the data provided. First, the authors nicely show that PLK1/LRRK1/NDEL1 are localized almost exclusively at the basal body, which makes it difficult to imagine how they would activate retrograde IFT at the ciliary tip. Second, the authors do not provide any evidence that NDEL1 physically interacts with dynein 2 or IFT complex components. Third, the results shown in Fig. 8 could equally well be explained by a requirement for PLK1/LRRK1/NDEL1 in promoting ciliary entry of dynein 2 or IFT complex proteins involved in retrograde IFT (e.g. some IFT-A proteins). In fact the latter model would be more compatible with the basal body localization of PLK1/LRRK1/NDEL1. Fourth, the authors need to consider and discuss their results/proposed model in the context of previously published work from the Snell lab, which showed that activation of axonemal disassembly in Chlamydomonas leads to increased entrance of empty IFT trains into cilia that possess cargo binding sites available to retrieve disassembled components (Pan and Snell, Dev Cell 9, pp 1-8, 2005). In sum, the authors need to provide additional evidence to confirm or refute their proposed model. Here are some suggestions for experiments that would strengthen the paper:

1) The authors could perform additional IFM analyses to investigate how depletion/inactivation of PLK1/LRRK1/NDEL1 affects ciliary localization of dynein 2 and IFT-A components, i.e. whether these fail to enter cilia and hence accumulate at the base.

2) Given that long-term depletion of DHC2 presumably leads to ciliary shortening, the cilia length in Fig. 7 needs to be quantified, and authors may consider supplementing these data with results from experiments where dynein activity is acutely inhibited by Ciliobrevin treatment.

3) If authors are able to do live cell imaging of IFT in RPE1 cells, they could consider testing how inhibition/depletion of PLK1/LRRK1/NDEL1 affects IFT dynamics.

Minor comments:

Page 4 bottom and page 5 top: the description of IFT is not accurate as IFT-A and IFT-B move together in trains in both anterograde and retrograde directions.
Please correct.

© 2022. Published by The Company of Biologists under the terms of the Creative Commons Attribution License (https://creativecommons.org/licenses/by/4.0/).

Page 14 middle: authors need to specify what they mean by “retrograde IFT proteins” since both IFT-A and IFT-B are moving bidirectionally inside cilia (see comment above).

Page 11 middle: the statement “Cilium resorption is thought to be required for the dynein-2-dependent transport of ciliary components from the tip to the base of the cilia” is not correct. Retrograde IFT occurs constitutively in many cilia and cell types and not only during cilia disassembly. Please correct text accordingly.

First revision

Author response to reviewers' comments

The manuscript has been revised in accordance with the comments raised by three Referees. Our responses to the comments are as follows:

Our responses to the comments of Reviewer #1

In 2011, Hanafusa et al. reported that GFP-LRRK1 localizes to early endosomes and regulates trafficking of EGFR. That is generally consistent with the major substrate of LRRK1 being Rab7, a late endosomal protein. They also reported that LRRK1 was needed for peripheral endosomes to be translocated to the perinuclear region. In 2015, Hanafusa et al. reported that LRRK1 is now instead localized exclusively to centrosomes throughout the cell cycle and may be phosphorylated by CDK1/cyclin B in vitro at position T1400. [Which is it? Please clarify with localization data here--for example Fig 1 and 2 are different and many micrographs are very hard to see].

In Fig. 1E, Myc-LRRK1 was expressed exogenously. Under this condition, LRRK1 was uniformly localized in the cytoplasm. However, Fig. 2A shows the localization of endogenous LRRK1 using an antibody against LRRK1. Under this condition, LRRK1 was localized to the centrosome throughout the cell cycle.

The authors used this finding to conclude that this modification "activates" LRRK1 but this was not shown and is a premature conclusion.

We have previously shown that Thr-1400 phosphorylation is required for the activation of LRRK1 kinase activity (Hanafusa et al., Nat. Cell Biol., 2015).

In most kinases, activation occurs through the phosphorylation of specific residues in the activation loop of the kinase domain. The Thr-1400 residue of LRRK1 is located in the activation loop. Therefore, it is reasonable that Thr-1400 phosphorylation activates the kinase activity of LRRK1.

The authors previously reported association of PLK1 with LRRK1 in mitosis and transphosphorylation at LRRK1 S1790. It is not yet clear how this modification influences LRRK1 activity or localization. It is also odd that this phosphosite has not been detected in multiple other lab's phosphoproteomics according to Phosphosite.com.

We have previously shown that PLK1-mediated phosphorylation of the LRRK1 Ser-1790 is required for CDK1-mediated Thr-1400 phosphorylation and the activation of LRRK1 (Hanafusa et al., Nat. Cell Biol., 2015). In the present study, using a PLK1 inhibitor, we showed that PLK1 phosphorylation of LRRK1 Ser-1790 is required for LRRK1 activation at the ciliary base (Fig. 2G, H) but not for its localization at the ciliary base (Fig. 2I).

It is hard to say why this phosphosite has not been detected in the phosphoproteomics of several other labs. Negative information does not tell us anything.

© 2022. Published by The Company of Biologists under the terms of the Creative Commons Attribution License (https://creativecommons.org/licenses/by/4.0/).

Here the authors claim that LRRK1 is “stimulated” at the mother centriole in response to serum re-stimulation but they have not yet shown this directly (page 5 line 14).

We rewrote this part (p. 5, lines 13-19).

There is no question that this manuscript reports a great deal of work but many of the conclusions are truly premature and need better documentation. For example, the specificity of the 3hosphor-specific antibodies is not included [can a LRRK1 inhibitor be used? Please show full blots of whole cell extracts and knockouts to demonstrate specificity].

Our anti-pS155-NDEL antibody did not work by immunoblotting. Therefore, we have characterized this antibody by immunostaining. In immunostaining experiments, we detected a signal recognized by the anti- pS155-NDEL1 antibody when GFP-LRRK1(Y944F) was co-expressed with wild-type NDEL1, but not with NDEL1(S155A) (Fig. 4F). These results confirm that the pS155-NDEL1 antibody specifically recognizes NDEL1 phosphorylated at Ser-155.

We have already demonstrated the specificity of anti-pT1400-LRRK1 and anti-pS1790-LRRK1 antibodies (Hanafusa et al., Nat. Cell Biol., 2015).

It was not stated how the authors knew which cells had received the siRNAs (even what percentage) and how they knew what they were scoring in the rescue experiments or how much the rescue constructs were over-expressed.

As suggested, we present the efficiency of siRNA transfection with Alexa555-labeled RNA oligos in RPE1 cells (Fig. S1A). Under our conditions, the transfection efficiency of siRNA is estimated to be about 90% (>500 cells counted).

As suggested, we provide information on how moderate expression was assessed in the “MATERIALS AND METHODS” section.

Also, given the prior connections to cell division, it is possible that the blockade observed is more related to a cell cycle arrest than a direct link to cilia retraction, especially if the authors need to score phenotypes after such long times of serum starvation.

It is known that primary cilia are transiently formed at the G1 phase as well as the G0 phase of the cell cycle. Because LRRK1 is required for cilia disassembly, LRRK1 depletion may inhibit ciliary resorption during cell cycle progression, resulting in increased cilia formation in the presence of serum (Fig. 1A, B).

Specific comments.

Page 7. Line 10. “To monitor LRRK1 activation we used an anti-pT1400 LRRK1 antibody”. It has not been shown that this form is actually “activated”.

We have previously shown that Thr-1400 phosphorylation is required for the activation of LRRK1 kinase activity (Hanafusa et al., Nat. Cell Biol., 2015).

In most kinases, activation occurs through the phosphorylation of specific residues in the activation loop of the kinase domain. The Thr-1400 residue of LRRK1 is located in the activation loop. Therefore, it is reasonable that Thr-1400 phosphorylation activates the kinase activity of LRRK1.

Page 7 Line 21. “...this [Ser1790] phosphorylation is required for the activation of LRRK1 at mitotic centrosomes. Again, the consequence of this modification is not yet known.

We are currently investigating the mechanism by which PLK1 activates LRRK1 kinase activity by phosphorylating LRRK1 Ser-1790. However, this topic is beyond the scope of the present study.

© 2022. Published by The Company of Biologists under the terms of the Creative Commons Attribution License (https://creativecommons.org/licenses/by/4.0/).

Page 8 Line 14 It may be true that CDK1 may phosphorylate Thr1400 after Ser 1790 but again, the consequence of these events is not yet established.

We showed that PLK1-mediated phosphorylation of LRRK1 Ser-1790 is dispensable for its localization at the ciliay base (Fig. 2I).

Figure 4 seeks to convince the reader that GFP-LRRK1 binds NDEL1b and not NDEL1a. The inputs do not appear equal and this reader could not determine if the blots were by LICOR or ECL—if ECL, they cannot be compared due to non-linearity of the method. The authors need to determine the significance of this interaction by generating a mutant that cannot bind and determining the consequences of a rescue with that protein.

We found that the NDEL1a form could also rescue the defect in cilia disassembly caused by NDEL1 knockdown. This result indicates that the strength of the interaction between LRRK1 and NDEL1 is not important for NDEL1 function. Therefore, we excluded the interaction results (Fig. 3).

Phosphorylation of NDEL1 is modest in vitro with large amounts of kinase and needs to be determined in cells at endogenous levels of enzyme and substrate. pS155 antibody needs to be properly validated to be sure it does not also recognize non-phospho or other site 4hosphor NDEL1.

In immunostaining experiments, we detected a signal recognized by the anti-pS155-NDEL1 antibody when GFP-LRRK1(Y944F) was co-expressed with wild-type NDEL1, but not with NDEL1(S155A) (Fig. 4F). These results confirm that the pS155-NDEL1 antibody specifically recognizes NDEL1 phosphorylated at Ser-155. Thus, the pS155-NDEL1 antibody does not recognize non-phospho NDEL1 or other sites of 5hosphor-NDEL1.

For all of these reasons, the story needs additional work before it is ready for presentation. It does appear that loss of LRRK1 increases the percent of ciliated cells but this may simply be due to a cell cycle arrest. The authors should use flow cytometry of cells with and without LRRK1 to check this possibility.

LRRK1 primarily promotes cilia disassembly, and its effect on cilia formation is considered a secondary effect. Notably, it is known that primary cilia are transiently formed at the G1 phase as well as the G0 phase of the cell cycle. Because LRRK1 is required for cilia disassembly, LRRK1 depletion may inhibit ciliary resorption during cell cycle progression, resulting in increased cilia formation in the presence of serum (Fig. 1A, B).

Thus, we agree with the comment that "It does appear that loss of LRRK1 increases the percent of ciliated cells but this may simply be due to a cell cycle arrest." In this study, we investigated how LRRK1 regulates cilia disassembly and did not focus on the mechanism by which loss of LRRK1 increases the percent of ciliated cells.

<u>**Our responses to the comments of Reviewer #2**</u>

Major points

1) Although the authors' findings are indeed provocative and clear, it is unclear to me how Ndel1 might affect dynein-2 activity from the ciliary base (i.e., distal from where dynein-2 functions)? I think potential mechanisms should at least be discussed.

One possibility is that LRRK1 phosphorylation of NDEL1 promotes the ciliary entry of dynein-2 or IFT complex proteins involved in retrograde IFT through interaction with the dynein-2 intermediate chains. We discuss this point (p. 15, lines 20-28 and p. 16, lines 1-20).

2) The authors' reasoning for focusing on Ndel1 is a bit unclear to me. They previously identified NudC as an LRRK1 binding partner. Why then follow- up with Ndel1 instead of NudC? Did the authors look into a potential role for NudC in ciliary function?

© 2022. Published by The Company of Biologists under the terms of the Creative Commons Attribution License (https://creativecommons.org/licenses/by/4.0/).

We focused on NDEL1 as a target for LRRK1 because we had reported that NDEL1 plays an important role in ciliary absorption upon cell cycle re-entry (Inaba et al., 2016). We rewrote this part (p. 8, lines 24-27).

3) Given the fact that Ndel1 binds to the unstructured N-terminus of the dynein-1 intermediate chain (McKenney et al., JBC 2011), the authors' findings suggest that Ndel1 might also bind to the N-terminus of the dynein-2 ICs (WRD60 or WRD34) and thereby affect its activity. This possibility should be discussed along with a description of any conservation between the IC N-termini.

As suggested, we have examined whether NDEL1 is associated with the dynein-2 intermediate chains, WDR34 and WDR60. We showed that NDEL1 is associated with WDR34 and WDR60 (Fig. 7). Furthermore, we found that WDR34 and WDR60 associate significantly less with non-phosphorylatable NDEL1(S155A) than wild-type NDEL1 or the S155D phosphomimic mutant (Fig. 7). These results indicate that Ser-155 phosphorylation of NDEL1 is critical for its interaction with dynein-2 intermediate chains.

4) Along these same lines, it is fairly well established that Ndel1 affects dynein-1 activity by affecting the dynein-LIS1 interaction. A previous study found that LIS1 may in fact localize to the cilium (Pederson et al., JCS 2007); however, there has been no follow-up or functional assessment of LIS1 in ciliary function. Although not a requirement for publication, I think it would be very interesting to simply test the role of LIS1 in ciliary resorption (and perhaps IFT88 transport, as shown in Figure 8). If LIS1 depletion does not cause the same phenotype as Ndel1 depletion, this would suggest a LIS1- independent function for Ndel1, which would be interesting. If Ndel1 does function via LIS1, then I would expect depletion of one or the other to cause similar phenotypes, whereas combined depletion should not be additive in severity.

Since LIS1 is not localized to non-motile cilia (Pedersen et al., J. Cell Sci., 2007) and is not associated with dynein-2 (Asante et al., J. Cell Sci.,), we have not examined the role of LIS1 in ciliary resorption.

Minor point:

1) "Our findings provide evidence for a link between LRRK1 and NDEL1 and implicate in the regulation of cilia disassembly via dynein-2-mediated retrograde IFT." This sentence is confusingly written. I think the authors meant: "Our findings provide evidence for a link between LRRK1 and NDEL1 and implicate these molecules in the regulation of cilia disassembly via dynein-2- mediated retrograde IFT."

As suggested, we have corrected this description.

<u>Our responses to the comments of Reviewer #3</u>

Main comments

Figure 7 and 8, p. 11-12 and Discussion: the authors show that DHC2- depleted cells fail to deciliate after 24 hr of serum re-addition (Fig. 7) and based on data indicating that IFT88 accumulates at the tip and base of these cells, as well as in cells depleted for LRRK1 or NDEL1 (Fig. 8), they propose that PLK1/LRRK1/NDEL1 promote cilia disassembly by activating dynein 2-mediated retrograde IFT at the ciliary tip. However, I find that this model is not well supported by the data provided.

First, the authors nicely show that PLK1/LRRK1/NDEL1 are localized almost exclusively at the basal body, which makes it difficult to imagine how they would activate retrograde IFT at the ciliary tip.

We agree with this comment. We have discussed this point (p. 15, lines 20– 28 and p. 16, lines 1-20).

© 2022. Published by The Company of Biologists under the terms of the Creative Commons Attribution License (https://creativecommons.org/licenses/by/4.0/).

Second, the authors do not provide any evidence that NDEL1 physically interacts with dynein 2 or IFT complex components.

Since NDEL1 binds to the dynein-1 intermediate chain, it was hypothesized that NDEL1 could also bind to the dynein-2 intermediate chains, WDR34 and WDR60, thereby affecting their activity. To test this possibility, we transfected Flag-NDEL1 into HEK293 cells with GFP-WDR34 or GFP- WDR60. We showed that NDEL1 bound to WDR34 and WDR60 (Fig. 7). Furthermore, WDR34 and WDR60 were associated significantly less with non-phosphorylatable NDEL1(S155A) than wild-type NDEL1 or the S155D phosphomimic mutant (Fig. 7). These results indicate that Ser-155 phosphorylation of NDEL1 is important for its interaction with dynein-2 intermediate chains.

Third, the results shown in Fig. 8 could equally well be explained by a requirement for PLK1/LRRK1/NDEL1 in promoting ciliary entry of dynein 2 or IFT complex proteins involved in retrograde IFT (e.g. some IFT-A proteins). In fact, the latter model would be more compatible with the basal body localization of PLK1/LRRK1/NDEL1.

We agree with this comment. We have discussed this point (p. 15, lines 20– 28 and p. 16, lines 1-20).

Fourth, the authors need to consider and discuss their results/proposed model in the context of previously published work from the Snell lab, which showed that activation of axonemal disassembly in Chlamydomonas leads to increased entrance of empty IFT trains into cilia that possess cargo binding sites available to retrieve disassembled components (Pan and Snell, Dev Cell 9, pp 1-8, 2005). In sum, the authors need to provide additional evidence to confirm or refute their proposed model.

As suggested, we discuss our results in the context of previously published work from the Snell lab. (p. 16, lines 20-26).

Here are some suggestions for experiments that would strengthen the paper:

1) The authors could perform additional IFM analyses to investigate how depletion/inactivation of PLK1/LRRK1/NDEL1 affects ciliary localization of dynein 2 and IFT-A components, i.e. whether these fail to enter cilia and hence accumulate at the base.

As suggested, we have analyzed the effect of LRRK1 or NDEL1 depletion on the localization of the IFT-A protein, IFT140, in cilia. After 48 h of serum starvation, IFT140 was found only at the ciliary base in control cells (Fig. S6A, B). However, the loss of LRRK1 or NDEL1 had little effect on the ciliary localization of IFT140 (Fig. S6A, B).

We further examined whether LRRK1 and NDEL1 affected the ciliary localization of dynein-2 using GFP-tagged DYNC2LI1 (dynein-2 intermediate chain 1). However, we could not detect the ciliary localization of GFP-DYNC2LI1 under our experimental conditions.

2) Given that long-term depletion of DHC2 presumably leads to ciliary shortening, the cilia length in Fig. 7 needs to be quantified, and authors may consider supplementing these data with results from experiments where dynein activity is acutely inhibited by Ciliobrevin treatment.

As suggested, we quantified the cilia length in the DHC2-depleted RPE1 cells (Fig. S4).

We showed that Ciliobrevin inhibited ciliary resorption (Fig. S5A). Furthermore, we showed that IFT88 accumulated at the ciliary tip in Ciliobrevin-treated cells (Fig. S5B, C). Thus, acute inhibition of dynein-2 activity in serum-starved conditions results in the accumulation of IFT material at the ciliary tips. This is consistent with the role of dynein-2 in retrograde ciliary transport.

3) If authors are able to do live cell imaging of IFT in RPE1 cells, they could consider testing how inhibition/depletion of PLK1/LRRK1/NDEL1 affects IFT dynamics.

© 2022. Published by The Company of Biologists under the terms of the Creative Commons Attribution License (https://creativecommons.org/licenses/by/4.0/).

As suggested, we performed live cell imaging of GFP-IFT88 in RPE1 cells. However, we could not observe the movement of GFP-IFT88 in the cilia.

Minor comments:

Page 4 bottom and page 5 top: the description of IFT is not accurate as IFT- A and IFT-B move together in trains in both anterograde and retrograde directions. Please correct.

As suggested, we have corrected this description (p. 4, lines 26-29 and p. 5, lines 1-2).

Page 14 middle: authors need to specify what they mean by "retrograde IFT proteins" since both IFT-A and IFT-B are moving bidirectionally inside cilia (see comment above).

We rewrote this part (p. 15, lines 20-24).

Page 11 middle: the statement "Cilium resorption is thought to be required for the dynein-2-dependent transport of ciliary components from the tip to the base of the cilia" is not correct. Retrograde IFT occurs constitutively in many cilia and cell types and not only during cilia disassembly. Please correct text accordingly.

As suggested, we have corrected this description (p. 15, lines 9-10).

Second decision letter

MS ID#: JOCES/2022/259999

MS TITLE: LRRK1-mediated NDEL1 phosphorylation promotes cilia disassembly via dynein-2-driven retrograde intraflagellar transport

AUTHORS: Hiroshi Hanafusa, Shin Kedashiro, Mako Gotoh, Ko-hei Saitoh, Hironori Inaba, Tomoki Nishioka, Kozo Kaibuchi, Masaki Inagaki, Naoki Hisamoto, and Kunihiro Matsumoto
ARTICLE TYPE: Research Article

We have now reached a decision on the above manuscript.

To see the reviewers' reports and a copy of this decision letter, please go to: https://submit-jcs.biologists.organd click on the 'Manuscripts with Decisions' queue in the Author Area. (Corresponding author only has access to reviews.)

As you will see, two reviewers gave favourable reports but raised some minor points that will require amendments to your manuscript. In contrast, reviewer 1 raises two very important points the first of which I feel needs to be addressed before the paper can be accepted. I hope that you will be able to carry these out because I would like to be able to accept your paper.

Please ensure that you clearly highlight all changes made in the revised manuscript. Please avoid using 'Tracked changes' in Word files as these are lost in PDF conversion.

I should be grateful if you would also provide a point-by-point response detailing how you have dealt with the points raised by the reviewers in the 'Response to Reviewers' box. Please attend to all of the reviewers' comments. If you do not agree with any of their criticisms or suggestions please explain clearly why this is so.

Reviewer 1

Advance summary and potential significance to field

See prior review

© 2022. Published by The Company of Biologists under the terms of the Creative Commons Attribution License (https://creativecommons.org/licenses/by/4.0/).

Comments for the author

I don't wish to stop publication of this work but my concerns remain as follows:

Given the author responses, I remain concerned about the specificity of the LRRK1 antibody that stains centrosomes, completely unlike exogenously expressed protein (from multiple papers from this team). The authors showed fluorescence images of LRRK1 si-RNA treated cells stained with their anti-LRRK1 antibodies in their 2015 Nature Cell Bio paper and only ~50% of the signal went away upon LRRK1 depletion. If so, how do you know what you are looking at--the "real" signal or the "remaining" signal without including LRRK1 siRNA in all studies? This is problematic and the discrepancy with their own other work should really be properly addressed.

As for activation of LRRK1, these scientists and others monitor increases in phosphoRab7 levels in actual enzyme activation assays. This should really be shown as the authors did not show activation concomitant with T1400 phosphorylation--I looked hard at the 2015 paper and I saw that T1400A is kinase inactive. This does not mean that pT1400 is "activated", it just means that T1400 is important at that site.

SEE AND PLEASE CITE doi: https://doi.org/10.1101/2022.06.09.495448

<u>Reviewer 2</u>

Advance summary and potential significance to field

The authors have addressed all of my concerns.

Comments for the author

The only thing I will point out is a typo in the first paragraph of the revised manuscript:

“The originates from the basal body, a modified mother centriole which…”

I believe “cilia” is missing from this sentence.

<u>Reviewer 3</u>

Advance summary and potential significance to field

The authors have addressed my main comments, and I find the manuscript has improved substantially. The authors nicely demonstrate, using cell biological and molecular approaches, the involvement of LRRK1-mediated NDEL1 phosphorylation in disassembly of primary cilia, likely by activating retrograde IFT. Thus the manuscript provides new molecular insight into the process of primary cilia disassembly. I recommend acceptance for publication provided that the authors address the following minor comments.

Comments for the author

Minor comments

Page 3 line 3: add “primary cilium” after The.

Page 3 line 5: add ”and pericentriolar material” after daughter centriole.

Page 4, 4 lines from bottom: replace operated with “assembled and maintained”.

Page 4, 3 lines from bottom: replace multisubunit complexes with “multisubunit cargo-binding complexes”.

© 2022. Published by The Company of Biologists under the terms of the Creative Commons Attribution License (https://creativecommons.org/licenses/by/4.0/).

Page 15, 7 lines from bottom: in addition to Ocbina et al. 2011, Goggolidou et al. 2014 (PMID: 25294941) should also be cited here.

Figure 6D: the symbols for the data points are not in the same size/format, some of the circles look too large.

Legend to Figure S1, S2 and S3: RPE1 is misspelled REP1.

Second revision

Author response to reviewers' comments

The manuscript has been revised in accordance with the comments raised by three Referees. Our responses to the comments are as follows:

Our responses to the comments of Reviewer #1

Given the author responses, I remain concerned about the specificity of the LRRK1 antibody that stains centrosomes, completely unlike exogenously expressed protein (from multiple papers from this team). The authors showed fluorescence images of LRRK1 si-RNA treated cells stained with their anti-LRRK1 antibodies in their 2015 Nature Cell Bio paper and only ~50% of the signal went away upon LRRK1 depletion. If so, how do you know what you are looking at--the "real" signal or the "remaining" signal without including LRRK1 siRNA in all studies? This is problematic and the discrepancy with their own other work should really be properly addressed.

We have previously shown that GFP-LRRK1 is diffusely distributed within cells and becomes localized to endosomes in response to EGF stimulation (Hanafusa et al., Nat. Commun., 2011). In addition, when endogenous LRRK1 is detected using the anti-LRRK1 antibody, it is predominantly present in centrosomes throughout the cell cycle (Fig. 2A) (Hanafusa et al., Nat. Cell Biol., 2015). One explanation for the difference in localization of exogenous and endogenous LRRK1 is that endogenous LRRK1 is more efficiently recruited to the centrosome than transiently expressed exogenous LRRK1. Consistent with this, moderately expressed exogenous LRRK1 is uniformly localized in the cytoplasm (Fig. 1E), but when exogenous LRRK1 is highly expressed, its centrosomal localization is also detected (Fig. 5F). Thus, the expression level of exogenous LRRK1 is important for its centrosomal localization.

As this reviewer noted, staining of LRRK1 siRNA-treated cells with the anti-LRRK1 antibody reduced the signal intensity of LRRK1 at the centrosome by about 60~70% compared to control cells (Hanafusa et al, Nat. Cell Biol.). This quantitative assay includes a background of fluorescence intensity. Therefore, the effect of LRRK1 siRNA on fluorescence intensity is much stronger. Indeed, LRRK1 siRNA treatment significantly reduced LRRK1 expression levels (Fig. S1B).

As for activation of LRRK1, these scientists and others monitor increases in phosphoRab7 levels in actual enzyme activation assays. This should really be shown as the authors did not show activation concomitant with T1400 phosphorylation--I looked hard at the 2015 paper and I saw that T1400A is kinase inactive. This does not mean that pT1400 is "activated", it just means that T1400 is important at that site.

This argument is not relevant to this manuscript.

Since the phosphorylation level of T1400 correlates with the kinase activity of LRRK1, the activation of LRRK1 can be monitored by the phosphorylation level of T1400 using the anti-pT1400 LRRK1 antibody.

© 2022. Published by The Company of Biologists under the terms of the Creative Commons Attribution License (https://creativecommons.org/licenses/by/4.0/).

SEE AND PLEASE CITE doi: https://doi.org/10.1101/2022.06.09.495448

We have cited this literature.

<u>Our responses to the comments of Reviewer #2</u>

The only thing I will point out is a typo in the first paragraph of the revised manuscript: "The originates from the basal body, a modified mother centriole which…" I believe "cilia" is missing from this sentence.

We have corrected this.

<u>Our responses to the comments of Reviewer #3</u>

Minor comments

Page 3 line 3: add "primary cilium" after The.

Page 3 line 5: add "and pericentriolar material" after daughter centriole.

Page 4, 4 lines from bottom: replace operated with "assembled and maintained".

Page 4, 3 lines from bottom: replace multisubunit complexes with "multisubunit cargo-binding complexes".

Page 15, 7 lines from bottom: in addition to Ocbina et al. 2011, Goggolidou et al. 2014 (PMID: 25294941) should also be cited here.

Figure 6D: the symbols for the data points are not in the same size/format, some of the circles look too large.

Legend to Figure S1, S2 and S3: RPE1 is misspelled REP1.

We thank this reviewer for pointing out errors in our manuscript. We have corrected them.

<u>Third decision letter</u>

MS ID#: JOCES/2022/259999

MS TITLE: LRRK1-mediated NDEL1 phosphorylation promotes cilia disassembly via dynein-2-driven retrograde intraflagellar transport

AUTHORS: Hiroshi Hanafusa, Shin Kedashiro, Mako Gotoh, Ko-hei Saitoh, Hironori Inaba, Tomoki Nishioka, Kozo Kaibuchi, Masaki Inagaki, Naoki Hisamoto, and Kunihiro Matsumoto
ARTICLE TYPE: Research Article

I am happy to tell you that your manuscript has been accepted for publication in Journal of Cell Science, pending standard ethics checks.

© 2022. Published by The Company of Biologists under the terms of the Creative Commons Attribution License (https://creativecommons.org/licenses/by/4.0/).
